# The Role of Essential Oils and Their Main Compounds in the Management of Cardiovascular Disease Risk Factors

**DOI:** 10.3390/molecules26123506

**Published:** 2021-06-09

**Authors:** Jorge M. Alves-Silva, Mónica Zuzarte, Henrique Girão, Lígia Salgueiro

**Affiliations:** 1 Univ Coimbra, Coimbra Institute for Clinical and Biomedical Research, Faculty of Medicine, 3000-548 Coimbra, Portugal; jmasilva@student.ff.uc.pt (J.M.A.-S.); mzuzarte@uc.pt (M.Z.); hmgirao@fmed.uc.pt (H.G.); 2 Univ Coimbra, Faculty of Pharmacy, 3000-548 Coimbra, Portugal; 3 Univ Coimbra, Center for Innovative Biomedicine and Biotechnology, 3000-548 Coimbra, Portugal; 4 Clinical Academic Centre of Coimbra, 3000-548 Coimbra, Portugal; 5 Univ Coimbra, Chemical Process Engineering and Forest Products Research Centre, Department of Chemical Engineering, 3030-790 Coimbra, Portugal

**Keywords:** cardiovascular, risk factors, hypertension, diabetes, dyslipidemia, essential oils

## Abstract

Cardiovascular diseases (CVDs) are a global health burden that greatly impact patient quality of life and account for a huge number of deaths worldwide. Despite current therapies, several side effects have been reported that compromise patient adherence; thus, affecting therapeutic benefits. In this context, plant metabolites, namely volatile extracts and compounds, have emerged as promising therapeutic agents. Indeed, these compounds, in addition to having beneficial bioactivities, are generally more amenable and present less side effects, allowing better patient tolerance. The present review is an updated compilation of the studies carried out in the last 20 years on the beneficial potential of essential oils, and their compounds, against major risk factors of CVDs. Overall, these metabolites show beneficial potential through a direct effect on these risk factors, namely hypertension, dyslipidemia and diabetes, or by acting on related targets, or exerting general cellular protection. In general, monoterpenic compounds are the most studied regarding hypotensive and anti-dyslipidemic/antidiabetic properties, whereas phenylpropanoids are very effective at avoiding platelet aggregation. Despite the number of studies performed, clinical trials are sparse and several aspects related to essential oil’s features, namely volatility and chemical variability, need to be considered in order to guarantee their efficacy in a clinical setting.

## 1. Introduction

**Figure 1:**
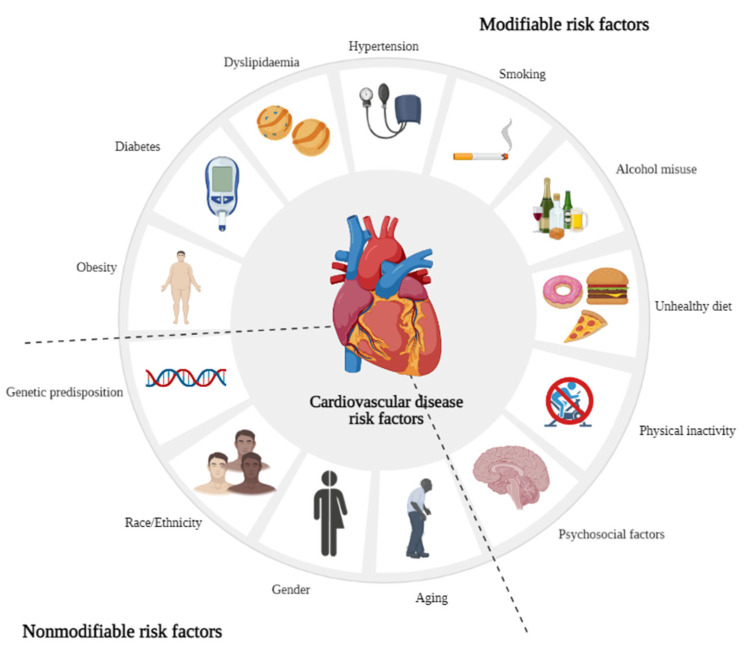
Cardiovascular disease risk factors. Created with BioRender.com.

Cardiovascular diseases (CVDs) continue to impact global health, as demonstrated by World Health Organization (WHO) reports, which show that CVDs account for 31% of total deaths worldwide [1]. The onset and progression of these disorders is highly dependent on several risk factors (Figure 1), aging being one of the most important. Moreover, by 2030, it is expected that 20% of the world´s population will be older than 65 years and CVDs will account for 40% of deaths in the elderly [2]. Besides aging, other non-modifiable risk factors, such as gender or genetic predisposition, play important roles in the onset of CVDs [1,3]. Furthermore, a family history of heart-related problems can lead to individuals developing CVDs, and genetic predisposition to other pathological conditions, such as type 2 diabetes, hypertension, or obesity increase the risk of CVD events [3]. Moreover, socioeconomic status and ethnicity are implicated in CVDs [3]. For example, individuals from African and Asian ethnicities have a higher risk of developing CVDs [4].

In addition to these risk factors, modifiable ones, such as hypertension, dyslipidemia, diabetes, obesity, smoking, alcohol misuse, unhealthy diet, sedentary lifestyle, and psychosocial factors are relevant and play determinant roles [5]; they are also included on the WHO target list to be reduced by 2025 [6]. The INTERHEART case-control study noted that 90% of acute myocardial infarction cases are due to these risk factors. Strikingly, controlling or eliminating them could, per se, lead to a drastic decrease in CVD mortality [7,8], strengthening the importance of new strategies to decrease the prevalence of these risk factors. 

It was reported that non-adherence to therapeutics occurs in 60% of CVD patients [9]. To decrease this trend, new therapeutic and/or preventive strategies with less side effects are imperative. In this scenario, natural products, particularly aromatic and medicinal plants, have emerged as promising agents to tackle cardiovascular disorders and associated risk factors. Despite the development of synthetic drugs, herbal medicines continue to be the source of basic healthcare for around 80% of the world’s population [10], thus pointing out their huge bioactive potential. Currently, herbs are used in the treatment of several chronic and acute conditions, including CVDs [11]. Their beneficial potential is also evidenced by the Mediterranean-style diet, which is embraced worldwide due to its reported health benefits, directly on CVDs or indirectly by reducing associated risk factors, such as cholesterol [12]. Furthermore, the European Medicines Agency (EMA) has 11 monographs approved for the use of herbal medicines in circulatory disorders [13]; thus, reinforcing their potential. The beneficial effects of herbal medicines are mainly attributed to their secondary metabolites [14], which are used in drug development, directly as therapeutic agents, or as starting materials and models for the synthesis of other drugs [11]. Secondary metabolites include phenolic compounds, terpenes, and alkaloids, among other classes [15]. Low molecular terpenes namely, monoterpenes (C_10_H_16_) and sesquiterpenes (C_15_H_24_) are the main compounds of essential oils. According to the International Standard Organization on Essential Oils (ISO 9235: 2013) [16] and the European Pharmacopoeia [17], an essential oil is defined as the product obtained from plant raw material by hydrodistillation, steam distillation, or dry distillation, or by a suitable mechanical process (for *Citrus* fruits). This definition excludes other aromatic products obtained by different extractive techniques, such as extraction with apolar solvents (concretes and absolutes). In some essential oils, phenylpropanoids, fatty acids, and their esters, as well as nitrogen and sulfur derivatives, are also present [18]. Bearing in mind the bioactive potential of these volatiles, the present review gathers a systematized compilation of the effects of essential oils and their compounds on major CVD risk factors, namely hypertension and dyslipidemia/diabetes. Moreover, other related beneficial effects are presented. In each section, a general consideration is included, followed by a compilation of the main studies, pointing out these effects. Then, mechanisms underlying the observed effects are referred, as well as the composition–activity relations reported in the cited paper or attempted by the authors of the present review. For this purpose, a bibliographic search was conducted using PubMed, Scopus, and Google Scholar databases, combining the keywords “essential oil”, “terpene” or “phenylpropanoid” with “cardiovascular”, “diabetes”, “obesity”, “dyslipidemia”, “hypertension” or “vasorelaxation”. Studies published over the last 20 years were considered; a total of 144 publications reporting these effects are included in the present review.

## 2. The Potential of Essential Oils and Their Compounds in the Management of Cardiovascular Diseases Risk Factors and Related Targets

### 2.1. Hypertension

#### 2.1.1. General Considerations

Hypertension mainly affects people from developed countries; its high prevalence (45% of general population) is attributed to poor lifestyle and behavioral habits, particularly diet, abusive consumption of alcohol, physical inactivity, and stress [19]. Elevated blood pressure is a red flag as it closely relates to an increased risk of heart disease [20]. Moreover, the majority of hypertensive patients concomitantly present other risk factors, increasing their risk of developing CVDs [21]. In the Framingham Heart Study, 80% of the enrolled hypertensive patients had at least one coexisting risk factor, whereas 55% of them had two or more risk factors [22]. These numbers are quite alarming, as it was shown that, in patients who have hypertension associated with other risk factors, the risk for CV events increases exponentially rather than the sum of individual risks [21]. Indeed, in prehypertensive individuals, the 10-year absolute risk for CVDs increases by 10%; however, when diabetes is also present, this risk increases by 40% [23].

Therapy relies on the use of drugs that usually control hypertension and decrease blood pressure; being the most frequently used diuretics, β-blockers, calcium antagonists, angiotensin converting enzyme (ACE) inhibitors, and angiotensin II receptor blockers (ARBs). However, approximately 35% of hypertensive patients discontinue their medication within 6 months, and in about 50% of the cases, adverse effects are present [24]. These facts reveal an urgent need for more effective and amenable antihypertensive agents that would increase patient compliance and reduce the socioeconomic burden associated with hypertension, mainly in developed countries.

#### 2.1.2. Hypotensive Essential Oils

Several studies show the antihypertensive potential of essential oils by assessing their effects in both normotensive and hypertensive pre-clinical models. In these models, hypertension is generally induced by deoxycorticosterone acetate (DOCA)-salt administration or nephrectomy. Moreover, since vasoconstriction is one of the major players associated with hypertension [25,26], the vasorelaxant effects of these extracts are frequently assessed. For vasorelaxation studies, ex vivo models are preferred, namely aortic rings (pre)contracted with different vasoconstrictor agents, such as phenylephrine (Phe) or high potassium concentrations. Table 1 summarizes the reported effects, with the studies being grouped according to the model used (in vitro, in vivo, or clinical trials). 

For the majority of the reported studies, the mechanisms by which the extracts exerted their beneficial effects were not disclosed. Nevertheless, in some cases, a more detailed study was performed, providing insight on possible underlying mechanisms. For example, the essential oils from *Croton zehntneri* induced hypotension that was abolished in the presence of capsaicin, a vanilloid receptor subtype 1 (TRPV1) inhibitor [77], suggesting that the essential oil might modulate this receptor´s activity [42]. The hypotensive and tachycardic effects reported for *Croton argyrophylloides* seem dependent on the parasympathetic nervous system, particularly on the muscarinic acetylcholine receptors, since both effects were reduced in the presence of methylatropine. In addition, the essential oil seems to act on the sympathetic system, especially on the nicotinic acetylcholine receptor, since the tachycardic effect was transformed into bradycardia upon hexamethonium pretreatment [67]. Similarly, the bradycardic effect of *Ocimum gratissimum* seems to depend on both parasympathetic and sympathetic systems since the effect was reduced by bilateral vagotomy or with methylatropine and hexamethonium, respectively [72]. Similar effects were observed for the essential oils of *Mentha x villosa* [48,49,68]. Furthermore, the effects observed on anesthetized rats treated with the essential oil from *Aniba rosaeodora* var. *amazonica* seems dependent on both the parasympathetic nervous system and vanilloid receptors since both effects were reduced by bilateral vagotomy or pretreatment with capsaicin, respectively. Opposingly, the administration of this oil to conscious rats was only dependent on the parasympathetic nervous system [64]. Similarly, the activity induced by the oil from *Aniba canelilla* is dependent on the parasympathetic nervous system, as well as on the nitric oxide (NO) axis [32]. *Artemisia campestris*’ essential oil seems to induce vasorelaxation via modulation of L-type Ca^2+^-channels and the activation of SERCA pumps [33]. The essential oil from *Pectis brevipedunculata* induces a vasorelaxant effect dependent on the NO/cyclic guanine monophosphate (cGMP) pathway since the pretreatment with L-NAME, an endothelial nitric oxide synthase (eNOS) inhibitor [78], decreased the observed relaxation [54]. The activity reported for the oil from *Trachyspermum ammi* is dependent on the extracellular Ca^2+^ flux, since pretreatment with nifedipine, a calcium channel blocker [79], reduced its activity [59]. The vasorelaxation induced by the essential oil from *Allium macrostemon* seems to be due to the phosphorylation of eNOS via intracellular Ca^2+^/protein kinase A (PKA)/eNOS pathway [27]. The activity of another oil characterized by sulfur-containing compounds, namely *Ferula asafoetida,* also appears to be dependent on NOS activity, since the presence of L-NAME partially abolished the reported effect. In addition, the activity seems to be mediated by prostaglandin activity since indomethacin, a COX inhibitor [80], reduced the vasorelaxation induced by the essential oil [44]. 

#### 2.1.3. Composition–Activity Relation

Essential oils are generally complex mixtures of several compound and it is known that their biological properties are, many times, due to synergistic effects between compounds [81] and/or the presence of active major/minor compounds. In this section, we present studies performed on isolated volatile compounds retrieved during the bibliographic search, in an attempt to identify putative active compounds present in the essential oils, and highlight possible composition–activity relations for the extracts compiled in Table 1. 

Several monoterpenes were studied in what concerns their hypotensive and vasorelaxant effects. For example, the enantiomeric isomers, (+)-α-pinene and (−)-β-pinene, were reported as inducers of hypotension associated with tachycardia [82]. Similarly, linalool, α-terpineol, and citronellol induced hypotension associated with tachycardia [83,84,85] and vasorelaxation [83,84,85,86,87]. Geraniol showed potential to treat arrhythmias via hypotensive and bradycardic effects [88]. Piperitenone oxide, 1,8-cineole and terpinen-4-ol caused hypotension and bradycardia [62,68,89,90]. These compounds were also reported as having vasorelaxant effects [91,92,93]. Carvacrol induced hypotension associated with bradycardia [94] and decreased heart rate, mean arterial pressure, as well as systolic and diastolic blood pressures [95]. In other studies, carvacrol and its isomer thymol induced vasorelaxation [94,96,97]. The same effect was reported for citral [54], linalyl acetate [98], carvone [86], and menthol [99,100]. Regarding sesquiterpenes, only vasorelaxant properties were reported. Indeed, caryophyllene oxide [87] and bisabolol [101,102] showed vasorelaxant effects using different contracting agents.

The hypotensive and vasorelaxant activities of phenylpropanoids were also widely reported. Indeed, estragole and anethole, induced hypotension associated with bradycardia in the first stage and hypertension with sustained bradycardia in late stages, in both conscious and normotensive rats [65]. Both compounds induced vasorelaxation [45,103]. Eugenol was greatly studied as a hypotensive agent [71,104,105]. Furthermore, several authors reported the vasorelaxant activity of this phenylpropanoid [86,97,103,104,105,106,107,108]. In addition, two derivatives of eugenol were described as having vasorelaxant activity, namely iso-eugenol [103] and methyl eugenol [40]. The vasorelaxant activity of cinnamaldehyde was widely reported [53,109,110] and similar effects were reported for methyl cinnamate [53,111].

For some of the essential oils compiled in Table 1, a composition–activity relation was highlighted. For example, the hypotensive effect reported for *Alpinia zerumbet* can be associated with the presence of high amounts of terpinen-4-ol and 1,8-cineole [62]. However, the vasorelaxant activity of this essential oil cannot be fully attributed to the presence of 1,8-cineole, since the compound elicits a full relaxation whereas the essential oil only elicited a partial one [29]; thus, suggesting an antagonistic effect of other compounds present in the mixture. Moreover, the hypotensive potential of *Mentha x villosa* essential oil is greater in samples with higher amounts of piperitenone oxide [68]; thus, suggesting that this compound is the main active compound in the essential oil. The monoterpene α-pinene was reported as a smooth muscle relaxant [112]; it may be responsible for the vasorelaxant effect observed for *Hyptis fruticosa* essential oil that presents high amounts of this compound [46]. Moreover, the vasorelaxant activity of *Citrus aurantium* var. *amara* can be explained by the presence of linalool, since this compound elicits a relaxant activity dependent on the NO/cGMP pathway [35]. The oil of bergamot (*Citrus bergamia*) also elicited a vasorelaxant effect that can be partially explained by the presence of linalool and linalyl acetate [36]. *Croton nepetaefolius* essential oil’s vasorelaxant activity might be due to the presence of 1,8-cineole and α-terpineol [40]. The reported activity of *Croton zehntneri* and *Foeniculum vulgare* is related to the presence of anethole and estragole, since both compounds were widely reported as having hypotensive and vasorelaxant activities [42,65,103]. Although eugenol was reported as having similar effects to those of *Ocimum gratissimum* oil, this volatile mixture also contains 1,8-cineole, which might contribute to the activity of the essential oil. The activity of *Ocotea quixos* oil can be attributed mainly to cinnamaldehyde, since it had a stronger activity than the whole essential oil. Contrarily, methyl cinnamate had a weaker activity than the extract [53]. *Allium macrostemon*’s major compound dimethyl trisulfide showed a vasoconstrictor activity whereas dimethyl disulfide had a preeminent vasodilator effect. Therefore, the activity described for *Allium macrostemon* is attributed mainly to dimethyl disulfide rather than to its major compound [27]. *Pectis brevipedunculata* exerted a vasorelaxant effect that may be attributed to the presence of citral, a mixture of neral and geranial, since these compounds alone are able to induce vasorelaxant effects, although to a lesser extent than that of the volatile extract [54]. In this case, the activity of the extract may have the contribution of geraniol, the other major compound of *P. brevipedunculata,* with both vasorelaxant and hypotensive activities reported [88].

### 2.2. Diabetes and Dyslipidemia

#### 2.2.1. General Considerations

Lipoprotein functions and/or levels associated with CVDs are often caused by a disturbance of lipid metabolism [113]. Although dyslipidemia includes a wide spectrum of lipids, the most widely studied (and implicated in CVDs) are the increased levels of total cholesterol (TC) and low-density lipoprotein cholesterol (LDL-C). Indeed, high saturated and trans-fat-diets lead to high levels of cholesterol and increase the risk of heart disease and stroke [114]. Furthermore, increased blood cholesterol, particularly low-density lipoprotein cholesterol (LDL-C) and non-high-density lipoprotein cholesterol (non-HDL-C) are associated with higher mortality and odds of atherosclerotic cardiovascular disease [115]. In the presence of other factors, such as high blood pressure and tobacco use, the cholesterol-associated risk increases [116,117,118,119,120,121,122]. Therefore, compounds that impact on the levels of these lipids, either by inhibiting their absorption in the gut, such as phytosterols that inhibit cholesterol’s metabolism [123], or by modulating the activity of lipid metabolism enzymes, such as 3-hydroxy-3-methyl-glutaryl-CoA (HMG-CoA), acyl CoA acyltransferase (ACAT), and sterol regulatory element-binding protein (SREBP) [124,125], are good candidates for lipid-lowering agents.

Another very important risk factor for CVDs is diabetes mellitus (DM), which is characterized by elevated blood glucose [122]. A meta-analysis showed that individuals with diabetes have a higher prevalence of CVDs when compared to non-diabetic ones [126], this risk being positively correlated with fasting blood glucose levels [127]. Indeed, in a 7-year follow-up, individuals with type 2 diabetes, with a history of acute myocardial infarction, had 42% death rate, whereas in cases where no history was found, this rate decreased to 15.4%. For non-diabetic individuals, these values were 15.9% and 2.1%, respectively [128]. Furthermore, diabetes also leads to an increase in free fatty acids (FFA) levels; thus, contributing to dyslipidemia [129]. Diabetes can be controlled through non-pharmacological approaches, including exercise, diet, and other lifestyle adaptations. In more severe cases, a pharmacological approach is required with the use of drugs that modulate glucose metabolism, such as metformin, glucagon-like peptide 1 (GLP1) receptor agonists, dipeptidyl peptidase-4 (DPP-4) inhibitors, or sodium glucose co-transporter 2 (SGLT2) inhibitors [122,130]. In the following section, the effect of essential oils on dyslipidemia and diabetes is presented together, as many studies address these risk factors in parallel.

#### 2.2.2. Antidiabetic and Anti-Dyslipidemic Essential Oils

The antidiabetic and anti-dyslipidemic potential of several essential oils were assessed, as summarized in Table 2. In vitro approaches generally use conditions that mimic diabetes or dyslipidemia, by treating cells with high glucose or oxidized LDL (oxLDL). Regarding in vivo studies, rats are the preferred animal model, to which streptozotocin (STZ) is administered to induce diabetes, and high fat or high cholesterol diets are used to represent the unhealthy western diet. Table 2 includes the major compounds of the essential oil as well as the different effects, organized in accordance to the type of assay used (in vitro, in vivo, and clinical trials).

To the best of our knowledge, only one study assessed the mechanism underlying the antidiabetic/anti-dyslipidemic effects of essential oils. Indeed, turmeric (*Curcuma longa*) essential oils seem to ameliorate the oxidative stress and liver dysfunction elicited by a high fat diet, through modulation of the peroxisome proliferator-activated receptor α, liver X receptor α, and associated genes involved in lipid metabolism and transport [139].

#### 2.2.3. Composition–Activity Relation

Several studies assessed the antidiabetic and/or anti-dyslipidemic potential of isolated compounds present in essential oils. For example, thymol was able to improve the lipid profile and blood glucose levels in mice with type 2 diabetes mellitus induced by a high fat diet [145]. Its isomer, carvacrol, had a similar effect in diabetic mice submitted to high fat diet, and in addition, an improvement in the associated inflammatory profile was observed [146]. Geraniol ameliorated the lipid profile on NIH *nu/nu* mice as well as the expression of receptors and enzymes associated with lipid metabolism [147]. In atherogenic diet-fed Syrian hamsters, geraniol had a similar effect [148]. The administration of camphene on hyperlipidemic rats improved their lipid profile [149]. Linalool seems to affect LDL metabolism by decreasing its oxidation, as well as increasing the affinity to LDL receptor [150]. β-Caryophyllene improved blood glucose, lipid profile, as well as the antioxidant system on streptozotocin-induced diabetes [151,152,153]. In rats fed high-fat/fructose diets, this terpene had a similar effect [154,155]. It also increased hemoglobin levels with an accompanying decrease in glycated hemoglobin and restored the activity of glycolytic and lipogenic enzymes [156]. These activities are associated with the binding to type 2-cannabinoid receptor (CB2R) and with the activation of Arf6, a small G protein, in a dose-dependent manner, promoting glucose-induced insulin secretion [157]. Similarly, thujone improved the lipid profile on alloxan-induced diabetes [158], as well as fasting blood glucose in streptozotocin-induced diabetes [159,160]. The antidiabetic potential of thujones can be attributed to the inhibition of GLUT4 translocation mediated by AMPK phosphorylation and to the restoration of the phosphorylation levels of Akt, GSK-3β, and glycogen synthase [159,160]. β-asarone also improved blood glucose levels and glucose tolerance in high fat diet-induced obesity in rats [161]. In the same model, this compound improved the lipid profile and the antioxidant defense system [162]. On methylisobutylxanthine, dexamethasone, insulin (MDI)-induced 3T3-L1 differentiation, β-asarone decreased lipid droplets in a dose-dependent manner, as well as the expression of differentiation markers, and improved the lipid profile [131]. Furthermore, this compound improved the lipid profile in cholesterol-fed rats and decreased the atherogenic index [163]. Eugenol greatly improved the lipid profile in atherogenic diet-fed rats. Furthermore, it ameliorated the activity of lipid metabolism-associated enzymes, namely HMG-CoA and lipase, and improved the antioxidant system [164]. Similar effects were observed on triton-induced hyperlipidemic rats [165] and microemulsions of eugenol were able to improve the lipid profile in high fructose-induced dyslipidemia [142]. Cinnamaldehyde decreased nitrotyrosine and ROS production by increasing the expression of Nrf2 with concomitant increase of associated antioxidant genes [166]. 

Some studies correlated the anti-dyslipidemic effect of essential oils with their main compounds. It was shown that linalool seems to be responsible for *Plantago asiatica* essential oil’s effect [135]. Moreover, the anti-dyslipidemic activity of *Pinus koraiensis* essential oil seems to be partially explained by the anti-dyslipidemic activity of camphene, its major compound, although the authors also suggest a synergistic effect with other compounds [134]. The reported activity for *Acorus calamus* might be attributed to the presence of β-asarone, since this compound had an activity similar to that of the essential oil in the same experimental model [131]. However, the observed effect might also be attributed to the presence of cinnamaldehyde, since this phenylpropanoid was reported as having anti-dyslipidemic effects [166]. This compound might also contribute to the activity of *Cinnamomum tamala* due to the high amount found in the essential oil. Similarly, the high amount of thujone found in the essential oil from *Salvia officinalis* might explain the antidiabetic effects reported, since this compound showed blood glucose lowering effects in STZ-induced diabetes [159,160]. Moreover, eugenol, widely reported as having anti-dyslipidemic effects [164,165], might be responsible for the activity observed for *Syzygium aromaticum* due to its high content in the essential oil.

### 2.3. Related Beneficial Effects of Essential Oils

#### 2.3.1. Antiplatelet Effect 

##### General Considerations

Platelet aggregation is fundamental in physiological conditions to prevent hemorrhaging. However, in pathological conditions, platelets can hyperaggregate leading to the formation of thrombus [167]. This hyperaggregability is caused by an overproduction of proaggregatory factors and/or a sub-production of antiaggregatory agents. Several risk factors for CVDs, such as hypertension, tobacco, and diabetes, can induce platelets hyperactivation [168]. This can lead to myocardial infarction and stroke [168,169,170]. To avoid this, antiplatelet drugs are used, namely acetylsalicylic acid, clopidogrel and glycoprotein IIb/IIIa inhibitors. Despite their wide use, the response of patients to therapy shows great variability due to gene polymorphisms as well as clinical and/or environmental factors [171]. Therefore, new antiplatelet aggregation agents are required to improve the overall response to therapy.

##### Essential Oils with Antiplatelet Effects

In this context, the majority of the studies assess the capacity of the essential oils to inhibit platelet aggregation induced by several clotting agents in platelet-rich plasma. Nevertheless, pre-clinical models of thromboembolism that allow assessing the capacity of the extract to prevent death and paralysis events have also been used, although in less extend. Table 3 summarizes the anticoagulant capacity of several essential oils, organized according to the type of studies performed. 

Overall, the essential oils in Table 3 are able to modulate the arachidonic acid cascade, since most of them inhibited platelet aggregation induced by arachidonic acid and collagen. However, other mechanisms also seem to play an important role since some of these extracts inhibited the aggregation induced by adenosine diphosphate (ADP), 4β-phorbol-12-myristate-13-acetate (PMA), and thromboxane A_2_ agonist, without showing a pro-hemorrhagic potential, unlike acetylsalicylic acid, a widely used anticoagulant drug [45,53].

##### Composition–Activity Relation

The antiplatelet effects of isolated volatile compounds are widely reported. Indeed, it has been shown that anethole is able to decrease platelet aggregation induced by arachidonic acid (AA) (IC_50_ = 9.7 μg/mL), collagen (IC_50_ = 8.1 μg/mL), ADP (IC_50_ = 54 μg/mL), and thromboxane receptor agonist U46619 (IC_50_ = 147 μg/mL), but failed to achieve 50% of inhibition in PMA-induced platelet aggregation (42% at 300 μg/mL). Furthermore, anethole was able to decrease the clot retraction induced by thrombin (IC_50_ = 169 μg/mL). Moreover, in an acute pulmonary thromboembolism animal model, this compound decreased the paralysis events by 83%, without showing a pro-hemorrhagic effect [45]. Hydroxychavicol inhibited platelet aggregation induced by AA and, to a lesser extent, that induced by collagen and thrombin. Furthermore, it decreased thromboxane B_2_ (TXB_2_) production induced by AA (IC_50_ = 0.91 μM), collagen (IC_50_ = 1.2 μM), and by thrombin (<20% of TXB_2_ production at 0.5 μM). In addition, it inhibited cyclooxygenase-1 (COX-1; IC_50_ = 79.8 μM) and cyclooxygenase-2 (COX-2; IC_50_ = 64.8 μM) activity and the AA-induced reactive oxygen species (ROS) production (IC_50_ = 11.1 μM). This compound also inhibited AA-induced (IC_50_ = 3.9 μM) and collagen-induced calcium mobilization. Furthermore, in an ex vivo model, hydroxychavicol inhibited platelet aggregation in platelet-rich plasma and delayed the platelet plug formation [174]. Eugenol inhibited COX-1 activity (IC_50_ = 59.3 μM) but had a very weak activity on COX-2 (19% at 500 μM) [174].

The antiaggregatory effects of *Foeniculum vulgare* seem to be due to the presence of high amounts of anethole in the oil, since this compound showed an activity similar to that of the whole oil [45].

#### 2.3.2. Ion Channel Modulator Effect

##### General Considerations

Calcium is relevant in several physiological and pathological situations in different organ systems [124]. In the cardiovascular system, calcium is a messenger in muscle contractility as well as in platelet aggregation. In addition, in some pathologies, the intracellular calcium release during diastole is impaired, thus decreasing the relaxation needed for the correct functioning of the heart [125]. Furthermore, high extracellular concentrations of this ion are associated with an increased risk of CVDs. Therefore, compounds that are able to maintain an adequate intracellular amount of calcium are important for a correct heart function. 

##### Essential Oils with Ion Channel Modulation Capacity

Studies assessing the effect of essential oils on ion channel modulation are scarce and only in vitro models were used. Table 4 compiles the few available studies on the capacity of essential oils to maintain calcium homeostasis.

In what concerns the mechanism underlying the calcium channels modulation effects, only one study addresses this topic. Indeed, the essential oil from *Citrus aurantium* L. var. *amara* seems to modulate intracellular Ca^2+^ concentration via inhibition of channel-mediated extracellular Ca^2+^ influx and store-operated Ca^2+^ release mediated by the ryanodine receptor (RyR) signaling pathway [35].

##### Composition–Activity Relation

The ion modulation activity of several isolated compounds was reported as well. Indeed, thymol and carvacrol inhibited the L-type Ca^2+^ current [177]. In addition, thymol suppressed the activity of Ca^2+^ and K^+^ channels [178] and triggered the release of Ca^2+^ from the sarcoplasmic reticulum while blocking the activity of Ca^2+^ pumps [179]. Similarly, carvacrol inhibited the Ca^2+^ influx by L-type Ca^2+^-channels [94] and increased the intracellular Ca^2+^ concentration [180]. Moreover, 1,8-cineole was able to decrease the contractility of left ventricular papillary muscles by reducing the sarcolemmal Ca^2+^ influx [91]. Linalool and linalyl acetate decreased Ca^2+^ influx [83,175]. β-Caryophyllene oxide, a sesquiterpenic compound, inhibited both Ca^2+^ and K^+^ currents [181] and eugenol inhibited the L-type Ca^2+^ current [177]. The same effect was also reported for cinnamaldehyde [182].

The effect of *Alpinia speciosa* is linked to the presence of 1,8-cineole. Nevertheless, terpinene-4-ol [183] and γ-terpinene [184] have caused relaxation in non-cardiac muscles in a Ca^2+^ dependent manner; thus, suggesting that these compounds might also contribute to the activity of the whole essential oil [28]. The effect of *Citrus aurantium* var. *amara* essential oil appears to be dependent on the presence of linalool, since the essential oil, similarly to the isolated compound, blocks Ca^2+^ influx [35]. *Citrus bergamia* ion channel modulation seems to be due to the presence of linalyl acetate; however, other compounds may play a role, since the isolated compound had a weaker activity compared to the essential oil [175].

#### 2.3.3. Other Beneficial Cardiovascular Effects

In addition to the reported effects of the essential oils on major modifiable risk factors for CVDs and related targets, other beneficial effects, such as the induction of cell proliferation under nefarious conditions, can also contribute to decrease the burden of CVDs. Therefore, other beneficial effects were considered, as compiled in Table 5. Almost all of the presented studies were carried out in vitro, with the exception of one that assessed the heart function in a pre-clinical model.

##### Composition–Activity Relation

To the best knowledge of the authors, no studies comparing the activity of the essential oils with that of the isolated compounds were conducted for the effects reported in Table 5. Therefore, this section will only present the reported activities of isolated volatile compounds present in essential oils.

For example, farnesol, an acyclic sesquiterpene alcohol, was able to decrease infarct size after ischemia/reperfusion (I/R) events and prevented cell death in isolated cardiomyocytes, after simulated I/R [186]. Carvacrol decreased rat aortic smooth muscle cells migration, and proliferation associated with platelet-derived growth factor (PDGF). Furthermore, it decreased ROS production and the phosphorylation of ERK1/2 and p38 MAPK. In addition, this compound also inhibited the outgrowth of aortic sprouts as well as neointima formation [187]. Borneol increased cell viability on hypoxia/reoxygenation-stimulated cardiomyocytes [188]. On an in vitro model of ischemia/reperfusion, eugenol increased cell viability of cardiomyocytes subjected to hypoxia/reoxygenation [188]. Eugenol reduced the acute cardiotoxicity elicited by doxorubicin [189] and on an isoproterenol-induced myocardial infarction model. This compound improved both hemodynamic function as well as histological markers associated with infarction [190]. These effects were also observed in isoproterenol-induced myocardial infarction animals after treatment with cinnamaldehyde or cinnamic acid [191]. On aortic banding-induced cardiac pressure overload, cinnamaldehyde improved heart function and decreased fibrosis. Furthermore, it normalized the expression of genes associated with hypertrophy (atrial and brain natriuretic peptides and β-myosin heavy chain) and prevented the activation of ERK1/2 [192]. On lipopolysaccharide (LPS)-stimulated rats, cinnamaldehyde improved cardiac function and decreased the inflammatory response [193]. α-Asarone treatment of angiotensin-II (Ang-II)-stimulated endothelial cells improved intracellular NO levels and decreased both ROS production and endothelial nitric oxide synthase (eNOS) phosphorylation [194]. 

## 3. Conclusions

**Figure 2:**
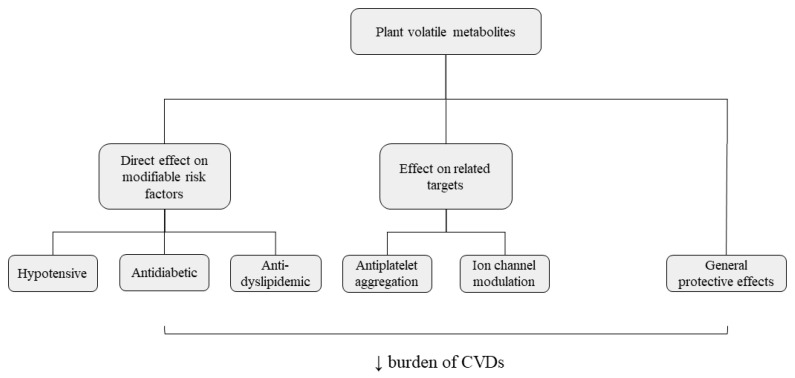
Role of plant volatiles (essential oils and isolated compounds) in the management of cardiovascular disease risk factors and associated targets.

The present review highlights the potential of essential oils and their compounds to decrease the burden of CVDs by targeting major associated risk factors and/or related targets. Despite the plethora of risk factors that lead to the development of CVDs, most of the studies using essential oils focus on hypertension, diabetes, and/or dyslipidemia/obesity. Nevertheless, other beneficial effects were also reported for these metabolites, namely avoidance of antiplatelet aggregation, modulation of ion channels, particularly calcium channels, as well as cellular protection against oxidative stress (Figure 2). Although, several studies described the beneficial effects for some volatile compounds, most of them did not attempt a composition–activity relation, and the activity of several compounds remain unknown, thus limiting their applicability. Overall, monoterpenic compounds were the most studied regarding their hypotensive as well as antidiabetic/anti-dyslipidemic effects, whereas phenylpropanoids exceled on counteracting platelet aggregation. The essential oils from *Alpinia* spp. stood out as the most effective due to their broad effects on both CVDs major risk factors and related ion channels activity. Moreover, the essential oils from the genus *Citrus* were very effective hypotensive agents, and those from *Foeniculum vulgare* showed both antidiabetic and antiplatelet aggregation effects. 

Although several in vitro and in vivo studies were performed over the last 20 years, clinical trials remain scarce and the majority focus on the hypotensive effects of essential oils. In these cases, the scientific name of the plant used, as well as its chemical characterization, are lacking, thus compromising a further exploitation for widespread use. In addition, small groups of individuals from the same region were recruited and, therefore, the genetic variability was not taken into account, thus jeopardizing a potential use in a clinical setting.

Overall, despite the huge potential of essential oils in decreasing the burden of CVDs, additional studies are needed. For example, important features of these extracts need to be considered, namely their high volatility and hydrophobicity, which can compromise bioavailability and consequent therapeutic outcomes. Moreover, the chemical variability among samples from the same *taxon* can compromise therapeutic efficacy. Indeed, in aromatic plants, the composition of essential oils may vary, depending on both intrinsic (seasonal, ontogenetic, and genetic variations and part of the plant used) and extrinsic (ecological and environmental aspects) factors. For this reason, standardized oils need to be guaranteed to avoid this kind of variability.

## Figures and Tables

**Table 1:** Hypotensive and vasorelaxant effects of essential oils.

Plant Species (Family)	Essential Oils Major Compounds	Study Model	Effect	Reference
**In Vitro Studies**				
*Allium macrostemon* Bunge (Amaryllidaceae)	Dimethyl trisulfide (34.93%), dimethyl disulfide (11.61%)	Isolated rat pulmonary arteries	Relaxation	[27]
*Alpinia speciosa* K. Schum (Zingiberaceae)	Terpinen-4-ol (38%), 1,8-cineole (18%), γ-terpinene (12%)	Rat left atria	↓ Force of contraction in a dose-dependent manner (IC_50_ = 292.2 µg/mL); ↓ sinus rhythm (IC_50_ = 595.4 µg/mL)	[28]
*Alpinia zerumbet* K. Schum (Zingiberaceae)	1,8-Cineole (33.3%), terpinen-4-ol (19.4%), p-cymene (11.4%)	Endothelium-intact rat aortic rings pre-contracted with Phe	Incomplete relaxation	[29]
	β-Phellandrene (16.4%), β-pinene (15.1%), 1,8-cineole (11%)	Rat aortic rings pre-contracted with norepinephrine and KCl	Inhibited contraction	[30]
*Aniba canelilla* (H.B.K.) Mez (Lauraceae)	EO without chemical characterization	Mesenteric arteries isolated from SHR	Relaxation on arteries contracted by K^+^ (IC_50_ = 294.19 µg/mL) or Phe (IC_50_ = 11.07 µg/mL); ↓ contractions evoked by phorbol butyrate and Phe in Ca^2+^-free medium; ↓ contractions induced by CaCl_2_ or BaCl_2_ in Ca^2+^-free and high K^+^ medium	[31]
		K^+^-induced contractions in rat aortic rings	IC_50_ = 64.5 µg/mL	[32]
*Artemisia campestris* L. (Asteraceae)	Spathulenol (10.2%), β-eudesmol (4.05%), p-cymene (3.83%)	Endothelium-intact rat aortic rings contracted with Phe	Contraction	[33]
*Citrusaurantifolia* (Christm) Swingle (Rutaceae)	Limonene (58.4%), β-pinene (15.4%)	Isolated rabbit aortic rings cultured in high K^+^ medium	Relaxation by activating Ca^2+^ channels	[34]
*Citrus aurantium* L. var. *amara* (Rutaceae)	Linalool (23.2%), β-pinene (9.6%), limonene (8.54%)	Endothelium-intact rat aortic rings pre-contracted with prostaglandin F_2α_	Vasorelaxation	[35]
*Citrus bergamia* Risso (Rutaceae)	D-Limonene (43.5%), linalyl acetate (25.5%)	Mouse aortic rings endothelium-intact and -denuded	Inhibited contraction elicited by PGF_2α_	[36]
*Croton argyrophylloides* Muell. Arg. (Euphorbiaceae)	Spathulenol (26.7%), caryophyllene oxide (13.1%), β-elemene (12.2%)	Endothelium-intact rat aortic rings and mesenteric arteries pre-contracted with Phe	Vasorelaxation on aortic rings (IC_50_ = 141.1 µg/mL) and mesenteric arteries (IC_50_ = 46.1 µg/mL)	[37]
*Croton nepetaefolius* Baill. (Euphorbiaceae)	1,8-Cineole (25.4%), bicyclogermacrene (11.1%)	Aortic rings isolated from DOCA-salt-hypertensive rats	↓ Contractions elicited by Phe	[38]
		RAT mesenteric vascular bed preparations	↓ Loss of flow caused by KCl	[39]
	1,8-Cineole (25.4%)	Endothelium-intact rat aortic rings	↓ Contractions evoked by KCl (IC_50_ = 26.7 µg/mL)	[40]
*Croton zambesicus* Müll. Arg. (Euphorbiaceae)	ent-Trachyloban-3-one (1.4–28.0%), caryophyllene oxide (2.9–25.9%), longifolene (0.4–26.4%)	Endothelium-intact rat aortic rings	Vasorelaxant activity (IC_50_ = 5.6–11.8 µg/mL)	[41]
*Croton zehntneri* Pax et Hoffm. (Euphorbiaceae)	Estragole (46%), *trans*-anethole (42.1%)	Endothelium-intact rat aortic preparations	↑ Phe-induced contractions (10 and 30 µg/mL); ↓ Phe-induced contractions (300–1000 µg/mL)	[42]
*Cymbopogon winterianus* Jowitt (Poaceae)	Geraniol (40.1%), citronellal (27.4%), citronellol (10.5%)	Rat mesenteric arteries contracted with KCl	Vasorelaxation on rings with (E_max_ = 125%) and without (E_max_ = 117%) endothelium; vasorelaxation in endothelium-denuded rings precontracted with KCl (E_max_ = 121%)	[43]
*Ferula asafoetida* L. (Apiaceae)	Di-(2-methyl-1,3-oxathiolanyl)methane (22.43%), trans-propenyl sec butyl disulfide (14.59%), 2-ethyltetrahydro- thiophene (10.61%), trans, trans-dibenzylideneacetone (10.07%)	K^+^-induced contractions in rat aortic rings	Relaxation on rings in the presence (IC_50_ = 1.6 µL/L) and absence (IC_50_ = 19.2 µL/L) of endothelium	[44]
*Foeniculum vulgare* Mill. (Apiaceae)	*trans*-Anethole (75.8%), Estragole (4.6%)	Phe-induced contractions in rat aortic rings	↓ Contractions on endothelium intact (IC_50_ = 108 μg/mL) and denuded (IC_50_ = 147 μg/mL) aortic rings	[45]
		K^+^-induced contractions in rat aortic rings	↓ Contractions on endothelium intact (IC_50_ = 64 μg/mL) and denuded (IC_50_ = 52 μg/mL) aortic rings	
*Hyptis fruticosa* Salzm. ex Benth (Lamiaceae)	α-Pinene, caryophyllene, 1,8-cineole	Endothelium-intact and -denuded rings from rat mesenteric artery pre-contracted with Phe	Relaxation (E_max_ = 64% and 122%, respectively); ↓ contractions induced by CaCl_2_ (E_max_ = 12% and 81%, respectively)	[46]
*Lippia thymoides* Mart. & Schauer (Verbenaceae)	β-Caryophyllene (26.3–17.2%)	Endothelium-intact and endothelium-denuded rat rings	Relaxation on endothelium-intact (IC_50_ = 305–544 µg/mL) and endothelium-denuded (IC_50_ = 150–283 µg/mL) rings	[47]
*Mentha x villosa* Huds. (Lamiaceae)	Piperitenone oxide (95.9%)	Isolated rat atrial preparations; Rat aortic rings	Dose-dependent negative chronotropic (IC_50_ = 229 µg/mL) and ionotropic (IC_50_ = 120 µg/mL) effects; Relaxation on aortic rings contracted with Phe- (IC_50_ = 255 µg/mL), PGF_2α_-induced (IC_50_ = 174 µg/mL) and KCl (IC_50_ = 165 µg/mL)	[48]
		Isolated rat aortic rings contracted by KCl	Relaxation (IC_50_ = 61 µg/mL and 109 µg/mL for endothelium-intact and denuded rings, respectively)	[49]
*Nigella sativa* L. (Ranunculaceae)	EO without chemical characterization	Intact rat aortic rings precontracted with noradrenaline and high K^+^	Vasorelaxation	[50]
*Ocimum gratissimum* L. (Lamiaceae)	Eugenol (43.7%)	Endothelium-intact rat aortic preparations	Vasorelaxation; ↓ Ca^2+^-induced contractions in Ca^2+^-free medium	[51]
	Eugenol (52.1%)	Endothelium-intact rat aortic rings	↓ Phe-induced contraction	[52]
		Rat mesenteric vascular beds	↓ Noradrenaline-induced perfusion pressure	
*Ocotea quixos* (Lam.) Kosterm. (Lauraceae)	*trans*-Cinnamaldehyde (27.8%), Methyl cinnamate (21.6%)	Rat aortic rings	↓ Phe-induced contractions on endothelium-intact (IC_50_ = 86 µg/mL) and endothelium-denuded (IC_50_ = 110 µg/mL) rings	[53]
*Pectis brevipedunculata* (Gardner) Sch. Bip. (Asteraceae)	Neral (32.7%), geranial (49.2%)	Phe-contracted rat aortic rings	Vasorelaxation on endothelium-intact (IC_50_ = 0.044%) and endothelium-denuded (IC_50_ = 0.093%) rings	[54]
*Psidium guajava* L. (Myrtaceae)	Butanoic acid methyl ester, 3-methyl glutaric anhydride, 1-butanol	Rat aortic rings	Vasorelaxation in aortic rings precontracted with Phe (EC_50_ = 6.23 mg/mL) and high K^+^ (EC_50_ = 5.52 mg/mL)	[55]
*Pogostemon elsholtzioides* Benth. (Lamiaceae)	Curzene (46.1%)	Rat aortic rings pre-contracted with Phe	Relaxation	[56]
*Rosa indica* L. (Rosaceae)	Methyl santonilate, butanoic acid, 2-methyl-5-oxo-1-cyclopentene-1-yl ester	Rat aortic rings	Vasorelaxation in aortic rings precontracted with high K^+^ (EC_50_ = 5.80 mg/mL) and Phe (EC_50_ = 7.39 mg/mL)	[57]
*Schinus areira* L. (Anacardiaceae)	α-Pinene (13.8%), limonene (12.8%), camphene (12.6%), β-caryophyllene (11.9%)	Ex vivo model of rabbit hearts	Inhibited the cardiac contractility induced by norepinephrine	[58]
*Trachyspermum ammi* (L.) Sprague (Apiaceae)	Thymol (38.1%), limonene (33.3%), p-cymene (23.1%)	Rat aortic rings	↓ Contractions of aortic rings induced by Phe (IC_50_ = 54.4 µg/mL), KCl (IC_50_ = 49 µg/mL) in the presence (IC_50_ = 46.6 µg/mL) and absence (IC_50_ = 45.2 µg/mL) of endothelium	[59]
*Xylopia langsdorfiana* A. St.-Hil. and Tul. (Annonaceae)	Germacrene D (22.9%), trans-β-guaiene (22.6%), β-caryophyllene (15.7%)	Isolated rat aortic rings contracted with Phe	Weak inhibition of contractions	[60]
**In Vivo Studies**				
*Alpinia zerumbet* K. Schum (Zingiberaceae)	Terpinen-4-ol (28.1%), 1,8-cineole (15.1%), γ-terpinene (13.7%)	Anesthetized and conscious rats	Hypotension	[61]
		Uninephrectomized normotensive rats	Hypotension	[62]
		DOCA-salt hypertensive rats	↓ MAP	
	Terpinene-4-ol (57.35%), 1,8-cineole (27.81%)	L-NAME-induced hypertensive rats	↓ MAP, SBP and DBP	[63]
*Aniba canelilla* (H.B.K.) Mez (Lauraceae)	1-Nitro-2-phenylethane (52.4%), methyl eugenol (38.6%)	Anesthetized and conscious rats	Hypotension with bradycardia	[32]
*Aniba rosaeodora* var. *amazonica* Ducke (Lauraceae)	(−)-Linalool (50.6%), (+)-linalool (49.4%)	Anesthetized rats	Hypotension with bradycardia	[64]
*Cymbopogon winterianus* Jowitt (Poaceae)	Geraniol (40.1%), citronellal (27.4%), citronellol (10.5%)	Conscious normotensive rats	Hypotension with tachycardia	[43]
*Croton zehntneri* Pax et Hoffm. (Euphorbiaceae)	Estragole (46%), *trans*-anethole (42.1%)	Conscious, normotensive rats	↓ MAP, ↓ HR (phase I); ↑ MAP, ↓ HR (phase II)	[65]
		Anesthetized, normotensive rats	Hypotension with bradycardia	[42]
		Conscious DOCA-salt hypertensive rats	↓ MAP, ↓ HR (phase I, 5–20 mg/kg); ↑ MAP, ↓ HR (phase II, 10, and 20 mg/kg)	[66]
*Croton argyrophylloides* Muell. Arg. (Euphorbiaceae)	Spathulenol (26.65%), caryophyllene oxide (13.13%), 𝛽-elemene (12.15%), 𝛽-caryophyllene (10.94%)	Conscious or anesthetized normotensive rats	Hypotension with tachycardia	[67]
*Hyptis fruticosa* Salzm., ex Benth (Lamiaceae)	α-Pinene, caryophyllene, 1,8-cineole	Non-anesthetized normotensive rats	Hypotension with tachycardia	[46]
*Mentha x villosa* Huds. (Lamiaceae)	Piperitenone oxide (95.9%)	DOCA-salt hypertensive rats	↓ MAP without bradycardia	[49]
			Hypotension and ↓ HR	[48]
	Piperitenone oxide (62.3%), γ-muurolene (16.0%)	Anesthetized rats	Hypotension with bradycardia	[68,69]
	Piperitenone oxide (95.9%)			
	Piperitenone oxide (55.4%), γ-muurolene (13.1%)	Normotensive conscious rats	↓ MAP and HR	[70]
*Ocimum gratissimum* L. (Lamiaceae)	Eugenol (43.7%), 1,8-cineole (32.7%)	Conscious DOCA-salt hypertensive rats	Hypotension	[51]
			Hypotension with bradycardia	[71]
		Uninephrectomized hypertensive rats	Hypotension with bradycardia	[71]
		Anesthetized or conscious, normotensive rats	↓ MAP, ↓ HR	[72]
*Pogostemon elsholtzioides* Benth. (Lamiaceae)	Curzene (46.1%)	Anesthetized rats	↓ SBP, DBP, MAP, and HR	[56]
*Schinus areira* L. (Anacardiaceae)	α-Pinene (13.8%), limonene (12.8%), camphene (12.6%)	Non-anesthetized normotensive rats	↓ SBP, DBP, and MAP	[58]
**Clinical Trials**				
Lavender (Lamiaceae)	EO without chemical characterization	Prehypertensive middle aged women	↓ SBP and DBP	[73]
Lavender (Lamiaceae):ylang ylang (Annonaceae):bergamot (Rutaceae) (5:3:2)	EO without chemical characterization	Individuals with essential hypertension	↓ SBP and DBP	[74]
Lavender (Lamiaceae)	EO without chemical characterization	Hypertensive individuals	↓ SBP 5-, 30- and 60-min post application ↓ DBP 60-min post application	[75]
Lavender (Lamiaceae):marjoram (Lamiaceae) (1:1)	EO without chemical characterization			
Lavender (Lamiaceae):marjoram (Lamiaceae):ylang-ylang (Annonaceae) (4:3:3)	EO without chemical characterization			
Lavender (Lamiaceae):ylang-ylang (Annonaceae):marjoram (Lamiaceae):neroli (Rutaceae) (20:15:10:2)	EO without chemical characterization	Pre- and hypertensive individuals	↓ Ambulatory BP (SBP (140.6 to 129.9 mmHg) and daytime DBP (90.5 to 83.3 mmHg)	[76]

BaCl_2_—barium chloride; CaCl_2_—calcium chloride; Ca^2+^—calcium ion; DBP—diastolic blood pressure; DOCA—deoxycorticosterone acetate; EC_50_ – half maximum effective concentration; E_max_—ventricular end-systolic maximum elastance; EO—essential oil; HR—heart rate; IC_50_—concentration needed to achieve 50% of relaxation; K^+^—potassium ion; KCl—potassium chloride; L-NAME—N(G)-nitro-L-arginine methyl ester; MAP—mean arterial pressure; PGF_2α_—prostaglandin F_2α_; Phe—phenylephrine; SBP—systolic blood pressure.

**Table 2:** Antidiabetic and anti-dyslipidemic essential oils.

Plant Species (Family)	Essential Oils Major Compounds	Study Model	Effect	References
**In Vitro Studies**				
*Acorus calamus* L. (Acoraceae)	β-Asarone (56.8%), eu-asarone (17.4%), cinnamaldehyde (4.7%)	MDI-induced 3T3-L1 differentiation	Prevents fat accumulation and preadipocytes differentiation into adipocytes	[131]
*Alpinia zerumbet* K. Schum (Zingiberaceae)	β-Phellandrene (16.4%), β-pinene (15.1%), 1,8-cineole (11%)	Human umbilical vessel endothelial cells (HUVECs)	↑ Cell viability in oxLDL-induced injury in HUVECs; ↓ LDH release (328.68 vs. 555.15 U/L) and MDA levels; ↑ GSH contents and ↑ SOD, CAT, GSH-Px activity	[132]
		Human aortic endothelial cells (HAECs) treated with oxLDL	↑ Cell viability; ↓ LDH release; ↑ MMP; ↓ ROS production; ↑ NO production; ↑ mRNA and protein levels of Akt/p-Akt, eNOS and sGC; ↓ iNOS levels	[133]
*Pinus koraiensis* Siebold and Zucc (Pinaceae)	Camphene (21.1%), D-limonene (21.0%), α-pinene (16.7%)	HepG2 cells	↑ mRNA and protein levels of LDL receptor; ↓ mRNA levels SREBP-1c, SREBP-2, HMG-CoA reductase, FAS and GPAT; ↓ activity of hACAT 1 and 2; ↓ oxidation of LDL	[134]
*Plantago asiatica* L. (Plantaginaceae)	Linalool (82.5%)	HepG2 cells	↑ LDL receptor; ↓ HMG-CoA reductase and LDL oxidation	[135]
*Salvia officinalis* L. (Lamiaceae)	α-Thujone (29%), 1,8-cineole (12%), β-caryophyllene (6.4%)	In vitro lipase and α-amylase activity inhibition	Inhibition of α-amylase (IC_50_ = 38 μg/mL) and lipase (IC_50_ = 52 μg/mL)	[136]
	*cis*-Thujone (17.4%), α-humulene (13.3%), 1,8-cineole (12.7%)	Primary normal hepatocytes growing in low glucose/lactate or in high glucose conditions	↓ Glucose production in normal hepatocytes; ↑ Glucose consumption on high glucose conditions in normal hepatocytes	[137]
**In Vivo** **Studies**				
*Cinnamomum tamala*, (Buch.-Ham.) Nees and Eberm (Lauraceae)	Cinnamaldehyde (44.9%), *trans*-cinnamyl acetate (25.3%)	STZ-induced type 2 diabetes rat model	↓ BG after 2h (280 and 239 vs. 341 mg/dL), 4h (292 and 272 vs. 332 mg/dL) and 28 days (201 and 201 vs. 410 mg/dL); ↓ BW loss (−5 and −10 g vs. −20 g); ↓ HbA1c (7.4 and 7.0 vs. 10.8% of Hb); ↑ hepatic glycogen (46 and 62 vs. 28 mg/g of tissue); ↑ insulin (9.8 and 12 vs. 7.8 µU/mL); ↓ TC (160 and 100 vs. 222 mg/dL); ↓ TG (28 and 20 vs. 40 mg/dL); ↑ HDL-C (45 and 52 vs. 36.4 mg/dL); ↓ MDA (4.0 and 3.2 vs. 5.2 nmol/dL); ↑ GSH (20 and 32 vs. 14 µmol GSH/g)	[138]
*Curcuma longa* L (Zingiberaceae)	ar-Turmerone (31.7%), β-turmerone (14.3%), α- turmerone (11.5%)	Golden Syrian hamsters consuming a high cholesterol diet	↓ TC, LDL-C and TG; ↑HDL-C in plasma (100 and 300 mg/kg); ↓ Hepatic TC, free cholesterol and cholesteryl ester	[139]
*Foeniculum vulgare* Mill. (Apiaceae)	EO without chemical characterization	STZ-induced diabetes rat model	↓ BG (81.97 vs. 162.5 mg/dL); ↑ GPx activity (99.60 vs. 59.72 U/g Hb)	[140]
		Diet-induced dyslipidemia	↓ BG (31 vs. 25% decrease); ↓ TC (81.62 vs. 97.43 mg/dL); ↑ HDL-C (40.6 vs. 37.18 mg/dL); ↓ LDL-C (11.09 vs. 21.31 mg/dL); ↓ TG (83.63 vs. 93.49 mg/dL); ↓ TNF-α (35.61 vs. 92.71 pg/mL); ↓ MDA (8.01 vs. 10.34 nmol/L); ↓ catalase (473.90 vs. 712.20 U/L); ↓ uric acid (7 vs. 7.5 mg/dL); ↓ plasma (0.36 vs. 0.38 mg/dL) and urinary (13.88 vs. 15.90 mg/dL) creatinine; ↓ urine volume (13.60 vs. 14.90 mL); ↓ creatinine clearance (0.37 vs. 0.50 mL/min); ↓ AST (35.80 vs. 44.79 U/L) and ALT (12.11 vs. 21.70 U/L)	[141]
*Plantago asiatica* L. (Plantaginaceae)	Linalool (82.5%)	C57BL/6 mice	↓ TC, TG levels; ↓ mRNA and protein levels of HMG-CoA reductase; ↑ mRNA of LDL receptor	[135]
*Salvia officinalis* L. (Lamiaceae)	α-Thujone (29%), 1,8-cineole (12%), β-caryophyllene (6.4%)	Alloxan-induced diabetes model	↓ α-Amylase activity by 47%; ↓ fasting blood glucose by 79%; ↑ hepatic glycogen by 44%; ↓ lipase by 53.3%; ↑ hepatic and renal function	[136]
*Syzygium aromaticum* (L.) Merrill and Perry [syn. *Eugenia caryophyllus* (Spreng.) Bullock and S. G. Harrison] (Myrtaceae)	Eugenol (75.2%)	High fructose-induced fatty liver and dyslipidemia in rats	Plasma: ↓ TC (147.7 vs. 164 mg/dL); ↓ TG (103.2 vs. 114.4 mg/dL); ↑ HDL-C (30.8 vs. 24.1 mg/dL); ↓ LDL-C (74 vs. 106.7 mg/dL); ↓ MDA (6.6 vs. 8.2 nmol/mL); ↓ TNF-α (25.5 vs. 31.9 pg/mL); ↓ ALT (72.5 vs. 85.7 U/L); ↓ AST (63.8 vs. 84.2 U/L); ↓ bilirubin (0.408 vs. 0.506 mg/dL) Liver: ↓ TF (35.4 vs. 46.0 mg/g tissue); ↓ TC (5.2 vs. 5.5 mg/g tissue); ↓ TG (8.8 vs. 9.4 mg/g tissue) ↓ body weight gain (72.6 vs. 83.1 g)	[142]
**Clinical trials**				
Cumin (Apiaceae)	EO without chemical characterization	Diabetic patients	↓ HbA1c (7.35 vs. 9.08%); ↓ FBG (116.4 vs. 181 mg/dL); ↓ TG (158.6 vs. 288 mg/dL); ↓ leptin (20.2 vs. 33.6 μg/mL); ↓ oxLDL (90.3 vs. 102.4 U/L); ↑ paraoxonase 1 (83.3 vs. 69.3 U/L); ↑ ApoA1 (115.4 vs. 97.7 mg/dL)	[143]
		Healthy individuals	↓ FBG by 55.9 mg/dL vs. 5.7 mg/dL in placebo; ↓ TNF-α by 1.38 ng/mL and CRP by 1.78 pg/mL; ↑ adiponectin by 57.11 μg/L)	[144]

AKT—protein kinase B; ALT—alanine aminotransferase; ApoA1—apolipoprotein A1; AST—aspartate aminotransferase; BG—blood glucose; BW—body weight; CAT—catalase; CRP—C-reactive protein; eNOS—endothelial nitric oxide synthase; EO—essential oil; FAS—fatty acid synthase; FBG—fasting blood glucose; GPAT—glycerol-3-phosphate acyltransferase; GSH—glutathione; GSH-Px—glutathione peroxidase; hACAT—human acyl CoA acyltransferase; Hb—hemoglobin; HbA1c—glycated hemoglobin; HDL-C—high density lipoprotein cholesterol; HMG-CoA—3-hydroxy-3-methyl-glutaryl-CoA; iNOS—inducible nitric oxide synthase; LDH—lactate dehydrogenase; LDL—low density lipoprotein; LDL-C—low density lipoprotein cholesterol; MDA—malondialdehyde; MDI—methylisobutylxanthine, dexamethasone, insulin; MMP—matrix metalloproteinase; NO—nitric oxide; oxLDL—oxidized LDL; pAKT—phosphorylated protein kinase B; ROS—reactive oxygen species; sGC—soluble guanylyl cyclase; SOD—superoxide dismutase; SREBP—sterol regulatory element-binding protein; STZ—streptozotocin; TC—total cholesterol; TF—total fat; TG—total triglycerides; TNF-α—tumor necrosis factor alpha.

**Table 3:** Essential oils with antiplatelet aggregation capacity.

Plant Species (Family)	Essential Oils Major Compounds	Study Model	Effect	References
**In Vitro** **Studies**				
*Artemisia dracunculus* L. (Asteraceae)	Estragole (70.1%)	ADP-, AA-, and U46619-induced platelet aggregation in guinea pig platelet-rich plasma	Inhibited platelet aggregation in a dose-dependent manner	[172]
		Thrombin-induced clot formation in guinea pig platelet-rich plasma	↓ Clot retraction in a dose-dependent manner (IC_50_ = 126 μg/mL)	
*Foeniculum vulgare* Mill. (Apiaceae)	*trans*-Anethole (75.8%), estragole (4.6%)	ADP-, AA- and U46619-, PMA- and collagen-induced platelet aggregation in guinea pig platelet-rich plasma	Inhibited platelet aggregation in a dose-dependent manner	[172]
			Inhibited ADP (IC_50_ = 50 μg/mL), AA (IC_50_ = 4.0 μg/mL), U46619 (IC_50_ = 132 μg/mL), PMA (46% at 300 μg/mL) and collagen (IC_50_ = 4.7 μg/mL) induced platelet aggregation	[45]
*Monarda didyma* L. (Lamiaceae)	Geraniol (89.5%)	Guinea pig and rat plasma	↓ AA-induced platelet aggregation (IC_50_ = 13 µg/mL)	[172]
*Ocimum basilicum* L. (Lamiaceae)	Linalool (49.9%)	Guinea pig and rat plasma	↓ AA-induced platelet aggregation (IC_50_ = 22 µg/mL)	[172]
*Ocotea quixos* (Lam.) Kosterm. (Lauraceae)	*trans-*Cinnamaldehyde (27.8), methyl cinnamate (21.6%)	ADP-, AA- and U46619-, PMA- and collagen-induced platelet aggregation in guinea pig platelet-rich plasma	Inhibited platelet aggregation in a dose-dependent manner	[172]
			Inhibited ADP (IC_50_ = 70 μg/mL), AA (IC_50_ = 47 μg/mL), U46619 (IC_50_ = 67 μg/mL), PMA (IC_50_ = 406 μg/mL) and collagen (IC_50_ = 163 μg/mL) induced platelet aggregation	[53]
		Thrombin-induced clot formation in guinea pig platelet-rich plasma	↓ Clot retraction in a dose-dependent manner (IC_50_ = 19 μg/mL)	[172]
		ADP- and U46619-induced platelet aggregation in human platelet-rich plasma	↓ ADP (IC_50_ = 128 μg/mL) and U46619 (IC_50_ = 115 μg/mL) induced aggregation	[53]
*Origanum vulgaris* L. (Lamiaceae)	Carvacrol (54.4%), thymol (14.3%)	Guinea pig and rat plasma	↓ AA-induced platelet aggregation (IC_50_ = 1.9 µg/mL)	[172]
*Syringa pinnatifolia* var. *alashanensis* (Oleaceae)	α-Cadinol (19.9%), α- muurolol (18.5%)	Primary cultured rat neonatal myocytes	↓ ADP-induced platelet aggregation	[173]
*Thymus vulgaris* L. (Lamiaceae)	p-Cymene (15.3%)	Guinea pig and rat plasma	↓ AA-induced platelet aggregation (IC_50_ = 4.7 µg/mL)	[172]
**In Vivo** **Studies**				
*Artemisia campestris* L. (Asteraceae)	Spathulenol (10.2%)	Wistar rats and albino mice	↓ Aggregation induced by thrombin (49.73% at 1 mg/mL) and ADP (48.20% at 1 mg/mL)	[33]
*Foeniculum vulgare* Mill. (Apiaceae)	*trans*-Anethole (75.8%), estragole (4.6%)	Acute pulmonary thromboembolism animal model	↓ Paralysis events (70% reduction at 30 μg/mL)	[45]
		Thrombin-induced clot formation	↓ Clot retraction in a dose-dependent manner (IC_50_ = 180 μg/mL)	[45,172]
*Ocotea quixos* (Lam.) Kosterm. (Lauraceae)	*trans-*Cinnamaldehyde (27.8%), methyl cinnamate (21.6%)	Acute pulmonary thromboembolism animal model	↓ Paralysis events (61% and 41% reduction at 100 and 30 μg/mL); ↓ death after 5 days (81% and 66% reduction at 100 µg/mL and 30 µg/mL)	[53]

AA—arachidonic acid; ADP—adenosine diphosphate; IC_50_—concentration required to achieve 50% inhibition of platelet aggregation; PMA—4β-phorbol-12-myristate-13-acetate; U46619 –thromboxane receptor agonist.

**Table 4:** Ion channel modulation by essential oils.

Plant Species (Family)	Essential Oils Major Compounds	Study Model	Effect	References
*Alpinia speciosa* K. Schum (Zingiberaceae)	Terpinen-4-ol (38%), 1,8-cineole (18%)	Whole-cell clamps	↓ Intercellular calcium (32.6% at 25 µg/mL vs. 89.3% at 250 µg/mL)	[28]
*Citrus aurantium* L. var. *amara* (Rutaceae)	Linalool (23.21%), β-pinene (9.59%), limonene (8.54%)	Smooth muscle cells	Relaxation caused by modulation of intracellular Ca^2+^	[35]
*Citrus bergamia* Risso (Rutaceae)	Limonene (43.5%), linalyl acetate (25.2%)	Mouse endothelial and vascular smooth muscle cells	Endothelial cells: Transient increase in intracellular Ca^2+^ followed by a decrease; Vascular smooth muscle cells: sustained ↑ intracellular calcium	[175]
*Nardostachys jatamansi* (D.Don) DC (Caprifoliaceae)	Calarene (38%), β-maaliene (7.9%), valerena-4,1(11)-diene (6.6%)	Human umbilical vein endothelial cells	↑ Intracellular Ca^2+^	[176]

Ca^2+^—Calcium ion.

**Table 5:** Beneficial cardiovascular effects of essential oils.

Plant Species (Family)	Essential Oils Major Compounds	Study Model	Effect	References
**In Vitro Studies**				
*Ocimum basilicum* L. (Lamiaceae)	Linalool (36–47.5%)	Primary cultures of cardiomyocytes treated with H_2_O_2_	↑ Cell proliferation	[185]
*Syringa pinnatifolia* Hemsl. (Oleaceae)	α-Cadinol (19.9%), τ-muurolol (18.5%)	Primary cultured rat neonatal myocytes	↓ H_2_O_2_-induced cell death	[173]
**In Vivo Studies**				
*Syringa pinnatifolia* Hemsl. (Oleaceae)	α-Cadinol (19.9%), τ-muurolol (18.5%)	Wistar rats, Kunming mice	↑ Survivability of rats under hypoxic conditions; ↓ Deviation on ST-segment; ↓ LDH, CK and TnT; ↑ SOD activity	[173]

CK—creatine kinase; H_2_O_2_—hydrogen peroxide; LDH—lactate dehydrogenase; SOD—superoxide dismutase; ST—segment and T-wave; TnT—cardiac troponin T.

## Data Availability

Data sharing not applicable.

## References

[1] WHO Cardiovascular Diseases. https://www.who.int/news-room/fact-sheets/detail/cardiovascular-diseases-(cvds).

[2] North B.J., Sinclair D.A. (2012). The intersection between aging and cardiovascular disease. Circ. Res..

[3] Buttar H.S., Li T., Ravi N. (2005). Prevention of cardiovascular diseases: Role of exercise, dietary interventions, obesity and smoking cessation. Exp. Clin. Cardiol..

[4] Leigh J.A., Alvarez M., Rodriguez C.J. (2016). Ethnic minorities and coronary heart disease: An update and future directions. Curr. Atheroscler. Rep..

[5] Mackay J., Mensah G.A. (2002). Risk factors. The Atlas of Heart Disease and Stroke.

[6] World Health Organization (2020). Noncommunicable Diseases: Campaign for Action—Meeting the NCD Targets.

[7] Timmis A., Townsend N., Gale C.P., Torbica A., Lettino M., Petersen S.E., Mossialos E.A., Maggioni A.P., Kazakiewicz D., May H.T. (2020). European Society of Cardiology: Cardiovascular disease statistics 2019. Eur. Heart J..

[8] Yusuf S., Hawken S., Ôunpuu S., Dans T., Avezum A., Lanas F., McQueen M., Budaj A., Pais P., Varigos J. (2004). Effect of potentially modifiable risk factors associated with myocardial infarction in 52 countries (the INTERHEART study): Case-control study. Lancet.

[9] Baroletti S., Dell’Orfano H. (2010). Medication adherence in cardiovascular disease. Circulation.

[10] Cordell G. (1995). Changing strategies in natural products chemistry. Phytochemistry.

[11] Wachtel-Galor S., Benzie I.F.F., Wachtel-Galor S., Benzie I.F.F. (2011). Herbal medicine: An introduction to its history, usage, regulation, current trends, and research needs. Herbal Medicine: Biomolecular and Clinical Aspects.

[12] Tuso P., Stoll S.R., Li W.W. (2015). A plant-based diet, atherogenesis, and coronary artery disease prevention. Perm. J..

[13] European Medicines Agency Herbal Medicines. https://www.ema.europa.eu/en/medicines/field_ema_web_categories%253aname_field/herbal/field_ema_herb_outcome/european-union-herbal-monograph-254/search_api_aggregation_ema_therapeutic_area_name/circulatorydisorders.

[14] Jenke-Kodama H., Müller R., Dittmann E. (2008). Evolutionary mechanisms underlying secondary metabolite diversity. Prog. Drug Res..

[15] Hartmann T. (2007). From waste products to ecochemicals: Fifty years research of plant secondary metabolism. Phytochemistry.

[16] ISO 9235 Aromatic Natural Raw Materials-Vocabulary 2013. https://www.iso.org/obp/ui/#iso:std:iso:9235:ed-2:v1:en.

[17] Council of Europe (2010). European Pharmacopoeia.

[18] Hüsnü K., Başer C., Demirci F., Berger R.G. (2007). Chemistry of essential oils. Flavours and Fragrances.

[19] Whelton P.K. (2002). Primary prevention of hypertension: Clinical and public health advisory from the national high blood pressure education program. JAMA.

[20] Kokubo Y., Iwashima Y. (2015). Higher blood pressure as a risk factor for diseases other than stroke and ischemic heart disease. Hypertension.

[21] Taddei S., Bruno R.M., Masi S., Solini A., Williams B. (2018). Epidemiology and pathophysiology of hypertension. ESC CardioMed.

[22] Kannel W. (2000). Risk stratification in hypertension: New insights from the Framingham study. Am. J. Hypertens..

[23] Egan B.M., Stevens-Fabry S. (2015). Prehypertension—Prevalence, health risks, and management strategies. Nat. Rev. Cardiol..

[24] Trinder Y. (2012). Common and less common adverse effects of antihypertensives: A general practitioner’s perspective. S. Afr. Fam. Pract..

[25] Giles T.D., Sander G.E., Nossaman B.D., Kadowitz P.J. (2012). Impaired vasodilation in the pathogenesis of hypertension: Focus on nitric oxide, endothelial-derived hyperpolarizing factors, and prostaglandins. J. Clin. Hypertens..

[26] Touyz R.M., Alves-Lopes R., Rios F.J., Camargo L.L., Anagnostopoulou A., Arner A., Montezano A.C. (2018). Vascular smooth muscle contraction in hypertension. Cardiovasc. Res..

[27] Han C., Qi J., Gao S., Li C., Ma Y., Wang J., Bai Y., Zheng X. (2017). Vasodilation effect of volatile oil from *Allium macrostemon* Bunge are mediated by PKA/NO pathway and its constituent dimethyl disulfide in isolated rat pulmonary arterials. Fitoterapia.

[28] Santos B.A., Roman-Campos D., Carvalho M.S., Miranda F.M.F., Carneiro D.C., Cavalcante P.H., Cândido E.A.F., Filho L.X., Cruz J.S., Gondim A.N.S. (2011). Cardiodepressive effect elicited by the essential oil of *Alpinia speciosa* is related to L-type Ca^2+^ current blockade. Phytomedicine.

[29] Pinto N.V., Assreuy A.M.S., Coelho-de-Souza A.N., Ceccatto V.M., Magalhães P.J.C., Lahlou S., Leal-Cardoso J.H. (2009). Endothelium-dependent vasorelaxant effects of the essential oil from aerial parts of *Alpinia zerumbet* and its main constituent 1,8-cineole in rats. Phytomedicine.

[30] Tao L., Hu H.S., Shen X.C. (2013). Endothelium-dependent vasodilatation effects of the essential oil from Fructus Alpiniae Zerumbet (EOFAZ) on rat thoracic aortic rings in vitro. Phytomedicine.

[31] Interaminense L.F.L., Dos Ramos-Alves F.E., de Siqueira R.J.B., Xavier F.E., Duarte G.P., Magalhães P.J.C., Maia J.G.S., Sousa P.J.D.C., Lahlou S. (2013). Vasorelaxant effects of 1-nitro-2-phenylethane, the main constituent of the essential oil of *Aniba canelilla*, in superior mesenteric arteries from spontaneously hypertensive rats. Eur. J. Pharm. Sci..

[32] Lahlou S., Magalhães P.J.C., de Siqueira R.J.B., Figueiredo A.F., Interaminense L.F.L., Maia J.G.S., da Sousa P.J.C. (2005). Cardiovascular effects of the essential oil of *Aniba canelilla* bark in normotensive rats. J. Cardiovasc. Pharmacol..

[33] Dib I., Fauconnier M.L., Sindic M., Belmekki F., Assaidi A., Berrabah M., Mekhfi H., Aziz M., Legssyer A., Bnouham M. (2017). Chemical composition, vasorelaxant, antioxidant and antiplatelet effects of essential oil of *Artemisia campestris* L. from Oriental Morocco. BMC Complement. Altern. Med..

[34] Spadaro F., Costa R., Circosta C., Occhiuto F. (2012). Volatile composition and biological activity of key lime *Citrus aurantifolia* essential oil. Nat. Prod. Commun..

[35] Kang P., Ryu K.H., Lee J.M., Kim H.K., Seol G.H. (2016). Endothelium- and smooth muscle-dependent vasodilator effects of *Citrus aurantium* L. var. amara: Focus on Ca2+ modulation. Biomed. Pharmacother..

[36] Kang P., Suh S.H., Min S.S., Seol G.H. (2013). The essential oil of *Citrus bergamia* Risso induces vasorelaxation of the mouse aorta by activating K^+^ channels and inhibiting Ca^2+^ influx. J. Pharm. Pharmacol..

[37] De França-Neto A., Cardoso-Teixeira A.C., Medeiros T.C., do Quinto-Farias M.S., de Sampaio C.M.S., Coelho-de-Souza A.N., Lahlou S., Leal-Cardoso J.H. (2012). Essential oil of *Croton argyrophylloides*: Toxicological aspects and vasorelaxant activity in rats. Nat. Prod. Commun..

[38] Lahlou S., Leal-Cardoso J.H., Magalhães P.J. (2000). Essential oil of *Croton nepetaefolius* decreases blood pressure through an action upon vascular smooth muscle: Studies in DOCA-salt hypertensive rats. Planta Med..

[39] Lahlou S., Leal-Cardoso J.H., Magalhães P.J.C., Coelho-de-Souza A.N., Duarte G.P. (1999). Cardiovascular effects of the essential oil of *Croton nepetaefolius* in rats: Role of the autonomic nervous system. Planta Med..

[40] Magalhães P.J.C., Lahlou S., Jucá D.M., Coelho-de-Souza L.N., da Frota P.T.T., da Costa A.M.G., Leal-Cardoso J.H. (2008). Vasorelaxation induced by the essential oil of *Croton nepetaefolius* and its constituents in rat aorta are partially mediated by the endothelium. Fundam. Clin. Pharmacol..

[41] Martinsen A., Baccelli C., Navarro I., Abad A., Quetin-Leclercq J., Morel N. (2010). Vascular activity of a natural diterpene isolated from *Croton zambesicus* and of a structurally similar synthetic trachylobane. Vascul. Pharmacol..

[42] De Siqueira R.J.B., Leal-Cardoso J., Couture R., Lahlou S. (2006). Role of capsaicin-sensitive sensory nerves in mediation of the cardiovascular effects of the essential oil of *Croton zehntneri* leaves in anaesthetized rats. Clin. Exp. Pharmacol. Physiol..

[43] De Menezes I.A.C., Moreira I.J.A., de Paula J.W.A., Blank A.F., Antoniolli A.R., Quintans-Júnior L.J., Santos M.R.V. (2010). Cardiovascular effects induced by *Cymbopogon winterianus* essential oil in rats: Involvement of calcium channels and vagal pathway. J. Pharm. Pharmacol..

[44] Esmaeili H., Sharifi M., Esmailidehaj M., Rezvani M.E., Hafizibarjin Z. (2017). Vasodilatory effect of asafoetida essential oil on rat aorta rings: The role of nitric oxide, prostacyclin, and calcium channels. Phytomedicine.

[45] Tognolini M., Ballabeni V., Bertoni S., Bruni R., Impicciatore M., Barocelli E. (2007). Protective effect of *Foeniculum vulgare* essential oil and anethole in an experimental model of thrombosis. Pharmacol. Res..

[46] Santos M.R.V., Carvalho A.A., Medeiros I.A., Alves P.B., Marchioro M., Antoniolli A.R. (2007). Cardiovascular effects of *Hyptis fruticosa* essential oil in rats. Fitoterapia.

[47] Silva F.S., Menezes P.M.N., de Sá P.G.S., de Oliveira A.L.S., Souza E.A.A., da Almeida J.R.G.S., de Lima J.T., Uetanabaro A.P.T., dos Silva T.R.S., Peralta E.D. (2016). Chemical composition and pharmacological properties of the essential oils obtained seasonally from *Lippia thymoides*. Pharm. Biol..

[48] Nunes Guedes D., Silva D.F., Barbosa-Filho J.M., Almeida De Medeiros I. (2004). Endothelium-dependent hypotensive and vasorelaxant effects of the essential oil from aerial parts of Mentha x villosa in rats. Phytomedicine.

[49] Lahlou S., Lima Carneiro-Leão R.F., Leal-Cardoso J.H. (2002). Cardiovascular effects of the essential oil of *Mentha x villosa* in DOCA-salt-hypertensive rats. Phytomedicine.

[50] Cherkaoui-Tangi K., Israili Z.H., Lyoussi B. (2016). Vasorelaxant effect of essential oil isolated from *Nigella sativa* L. seeds in rat aorta: Proposed mechanism. Pak. J. Pharm. Sci..

[51] Interaminense L.F.L., Jucá D.M., Magalhães P.J.C., Leal-Cardoso J.H., Duarte G.P., Lahlou S. (2007). Pharmacological evidence of calcium-channel blockade by essential oil of *Ocimum gratissimum* and its main constituent, eugenol, in isolated aortic rings from DOCA-salt hypertensive rats. Fundam. Clin. Pharmacol..

[52] Pires A.F., Madeira S.V.F., Soares P.M.G., Montenegro C.M., Souza E.P., Resende A.C., Soares de Moura R., Assreuy A.M.S., Criddle D.N. (2012). The role of endothelium in the vasorelaxant effects of the essential oil of *Ocimum gratissimum* in aorta and mesenteric vascular bed of rats. Can. J. Physiol. Pharmacol..

[53] Ballabeni V., Tognolini M., Bertoni S., Bruni R., Guerrini A., Rueda G.M., Barocelli E. (2007). Antiplatelet and antithrombotic activities of essential oil from wild *Ocotea quixos* (Lam.) Kosterm. (*Lauraceae*) calices from Amazonian Ecuador. Pharmacol. Res..

[54] Pereira S.L., Marques A.M., Sudo R.T., Kaplan M.A.C., Zapata-Sudo G. (2013). Vasodilator activity of the essential oil from aerial parts of *Pectis brevipedunculata* and its main constituent citral in rat aorta. Molecules.

[55] Rasheed H.M., Khan T., Wahid F., Khan R., Shah A.J. (2016). Chemical composition and vascular and intestinal smooth muscle relaxant effects of the essential oil from *Psidium guajava* fruit. Pharm. Biol..

[56] Shiva Kumar A., Jeyaprakash K., Chellappan D.R., Murugan R. (2017). Vasorelaxant and cardiovascular properties of the essential oil of *Pogostemon elsholtzioides*. J. Ethnopharmacol..

[57] Rasheed H.M., Khan T., Wahid F., Khan R., Shah A.J. (2015). Chemical composition and vasorelaxant and antispasmodic effects of essential oil from *Rosa indica* L. petals. Evid. Based Complement. Altern. Med..

[58] Bigliani M.C., Rossetti V., Grondona E., Lo Presti S., Paglini P.M., Rivero V., Zunino M.P., Ponce A.A. (2012). Chemical compositions and properties of *Schinus areira* L. essential oil on airway inflammation and cardiovascular system of mice and rabbits. Food Chem. Toxicol..

[59] Zadeh G.S., Panahi N. (2017). Endothelium-independent vasorelaxant activity of *Trachyspermum ammi* essential oil on rat aorta. Clin. Exp. Hypertens..

[60] De Correia A.C., Ferreira T.F., Martins I.R.R., Macêdo C.L., de Monteiro F., Costa V.C.O., Tavares J.F., Silva M.S., Paredes-Gamero E.J., Buri M.V. (2015). Essential oil from the leaves of *Xylopia langsdorfiana* (*Annonaceae*) as a possible spasmolytic agent. Nat. Prod. Res..

[61] Lahlou S., Galindo C.A.B., Leal-Cardoso J.H., Fonteles M.C., Duarte G.P. (2002). Cardiovascular effects of the essential oil of *Alpinia zerumbet* leaves and its main constituent, terpinen-4-ol, in rats: Role of the autonomic nervous system. Planta Med..

[62] Lahlou S., Interaminense L.F.L., Leal-Cardoso J.H., Duarte G.P. (2003). Antihypertensive effects of the essential oil of *Alpinia zerumbet* and its main constituent, terpinen-4-ol, in DOCA-salt hypertensive conscious rats. Fundam. Clin. Pharmacol..

[63] Da Cunha G.H., de Moraes M.O., Fechine F.V., Frota Bezerra F.A., Silveira E.R., Canuto K.M., de Moraes M.E.A. (2013). Vasorelaxant and antihypertensive effects of methanolic fraction of the essential oil of *Alpinia zerumbet*. Vascul. Pharmacol..

[64] De Siqueira R.J., Rodrigues K.M.S., da Silva M.T.B., Correia Junior C.A.B., Duarte G.P., Magalhães P.J.C., dos Santos A.A., Maia J.G.S., da Cunha P.J.S., Lahlou S. (2014). Linalool-rich rosewood oil induces vago-vagal bradycardic and depressor reflex in rats. Phyther. Res..

[65] De Siqueira R.J.B., Magalhães P.J.C., Leal-Cardoso J.H., Duarte G.P., Lahlou S. (2006). Cardiovascular effects of the essential oil of *Croton zehntneri* leaves and its main constituents, anethole and estragole, in normotensive conscious rats. Life Sci..

[66] De Siqueira R.J.B., Duarte G.P., Magalhães P.J.C., Lahlou S. (2013). Cardiovascular effects of the essential oil of *Croton zehntneri* leaves in DOCA-salt hypertensive, conscious rats. Nat. Prod. Commun..

[67] Alves-Santos T.R., de Siqueira R.J.B., Duarte G.P., Lahlou S. (2016). Cardiovascular effects of the essential oil of *Croton argyrophylloides* in normotensive rats: Role of the autonomic nervous system. Evid. Based Complement. Altern. Med..

[68] Lahlou S., Carneiro-Leão R.F.L., Leal-Cardoso J.H., Toscano C.F. (2001). Cardiovascular effects of the essential oil *Mentha x villosa* and its main constituent, piperitenone oxide, in normotensive anaesthetised rats: Role of the autonomic nervous system. Planta Med..

[69] Lahlou S., Magalhães P.J.C., Carneiro-Leão R.F.L., Leal-Cardoso J.H. (2002). Involvement of nitric oxide in the mediation of the hypotensive action of the essential oil of *Mentha x villosa* in normotensive conscious rats. Planta Med..

[70] Matos F.J.D.A., Machado M.I.L., Craveiro A.A., Alencar J.W., Barbosa J.M., da Cunha E.V.L., Hiruma C.A. (1999). Essential oil of *Mentha x villosa* Huds. from Northeastern Brazil. J. Essent. Oil Res..

[71] Interaminense L.F.L., Leal-Cardoso J.H., Magalhães P.J.C., Duarte G.P., Lahlou S. (2005). Enhanced hypotensive effects of the essential oil of *Ocimum gratissimum* leaves and its main constituent, eugenol, in DOCA-salt hypertensive conscious rats. Planta Med..

[72] Lahlou S., Interaminense Lde F., Leal-Cardoso J.H., Morais S.M., Duarte G.P. (2004). Cardiovascular effects of the essential oil of *Ocimum gratissimum* leaves in rats: Role of the autonomic nervous system. Clin. Exp. Pharmacol. Physiol..

[73] Jung Y.J. (2007). Effects of Aromatherapy on Blood Bressure, Heart Rate Variability, and Serum Catecholamines in the Pre-Hypertension Middle Aged Women. Ph.D. Thesis.

[74] Hwang J.H. (2006). The effects of the inhalation method using essential oils on blood pressure and stress responses of clients with essential hypertension. Taehan. Kanho. Hakhoe. Chi..

[75] Jang H.H. (2002). A clinical study on the effects of the aromatherapy for hypertension. J. Orient Neuropsychiatry.

[76] Kim I.H., Kim C., Seong K., Hur M.H., Lim H.M., Lee M.S. (2012). Essential oil inhalation on blood pressure and salivary cortisol levels in prehypertensive and hypertensive subjects. Evid. Based Complement. Altern. Med..

[77] Story G.M., Cruz-Orengo L. (2007). Feel the burn. Am. Sci..

[78] Kopincová J., Púzserová A., Bernátová I. (2012). L-NAME in the cardiovascular system–nitric oxide synthase activator?. Pharmacol. Reports.

[79] Mcdonald T.F., Pelzer S., Trautwein W., Pelzer D.J. (1994). Regulation and modulation of calcium channels in cardiac, skeletal, and smooth muscle cells. Physiol. Rev..

[80] Ferreira S.H., Moncada S., Vane J.R. (1971). Indomethacin and aspirin abolish prostaglandin release from the spleen. Nat. New Biol..

[81] Bassolé I.H.N., Juliani H.R. (2012). Essential oils in combination and their antimicrobial properties. Molecules.

[82] Menezes I.A.C., Barreto C.M.N., Antoniolli A.R., Santos M.R.V., de Sousa D.P. (2010). Hypotensive activity of terpenes found in essential oils. Z. Naturforsch. C..

[83] Anjos P.J.C., Lima A.O., Cunha P.S., De Sousa D.P., Onofre A.S.C., Ribeiro T.P., Medeiros I.A., Antoniolli Â.R., Quintans-Júnior L.J., Santos M.R.V. (2013). Cardiovascular effects induced by linalool in normotensive and hypertensive rats. Z. Naturforsch. Sect. C J. Biosci..

[84] Bastos J.F.A., Moreira Í.J.A., Ribeiro T.P., Medeiros I.A., Antoniolli A.R., De Sousa D.P., Santos M.R. (2010). V Hypotensive and vasorelaxant effects of citronellol, a monoterpene alcohol, in rats. Basic Clin. Pharmacol. Toxicol..

[85] Ribeiro T.P., Porto D.L., Menezes C.P., Antunes A.A., Silva D.F., De Souza D.P., Nakao L.S., Braga V.A., Medeiros I.A. (2010). Unraveling the cardiovascular effects induced by α-terpineol: A role for the NO-cGMP pathway. Clin. Exp. Pharmacol. Physiol..

[86] Kundu S., Shabir H., Basir S.F., Khan L.A. (2014). Inhibition of As(III) and Hg(II) caused aortic hypercontraction by eugenol, linalool and carvone. J. Smooth Muscle Res..

[87] Baccelli C., Martinsen A., Morel N., Quetin-Leclercq J. (2010). Vasorelaxant activity of essential oils from *Croton zambesicus* and some of their constituents. Planta Med..

[88] De Menezes-Filho J.E.R., Gondim A.N.S., Cruz J.S., de Souza A.A., Dos Santos J.N.A., Conde-Garcia E.A., de Sousa D.P., Santos M.S., de Oliveira E.D., de Vasconcelos C.M.L. (2014). Geraniol blocks calcium and potassium channels in the mammalian myocardium: Useful effects to treat arrhythmias. Basic Clin. Pharmacol. Toxicol..

[89] Guedes D.N., Silva D.F., Barbosa-Filho J.M., Medeiros I.A. (2002). Muscarinic agonist properties involved in the hypotensive and vasorelaxant responses of rotundifolone in rats. Planta Med..

[90] Lahlou S., Figueiredo A.F., Magalhães P.J.C., Leal-Cardoso J.H. (2002). Cardiovascular effects of 1,8-cineole, a terpenoid oxide present in many plant essential oils, in normotensive rats. Can. J. Physiol. Pharmacol..

[91] Soares M.C.M.S., Damiani C.E.N., Moreira C.M., Stefanon I., Vassallo D.V. (2005). Eucalyptol, an essential oil, reduces contractile activity in rat cardiac muscle. Braz. J. Med. Biol. Res. Rev. Bras. Pesqui. Med. Biol..

[92] Guedes D.N., Silva D.F., Barbosa-Filho J.M., Medeiros I.A. (2004). Calcium antagonism and the vasorelaxation of the rat aorta induced by rotundifolone. Braz. J. Med. Biol. Res..

[93] Maia-Joca R.P.M., Joca H.C., Ribeiro F.J.P., Do Nascimento R.V., Silva-Alves K.S., Cruz J.S., Coelho-De-Souza A.N., Leal-Cardoso J.H. (2014). Investigation of terpinen-4-ol effects on vascular smooth muscle relaxation. Life Sci..

[94] Dantas B.P.V., Alves Q.L., de Assis K.S., Ribeiro T.P., de Almeida M.M., de Vasconcelos A.P., de Araújo D.A.M., de Andrade Braga V., de Medeiros I.A., Alencar J.L. (2015). Participation of the TRP channel in the cardiovascular effects induced by carvacrol in normotensive rat. Vascul. Pharmacol..

[95] Aydin Y., Kutlay Ö., Ari S., Duman S., Uzuner K., Aydin S. (2007). Hypotensive effects of carvacrol on the blood pressure of normotensive rats. Planta Med..

[96] Peixoto-Neves D., Silva-Alves K.S., Gomes M.D.M., Lima F.C., Lahlou S., Magalhães P.J.C., Ceccatto V.M., Coelho-De-Souza A.N., Leal-Cardoso J.H. (2010). Vasorelaxant effects of the monoterpenic phenol isomers, carvacrol and thymol, on rat isolated aorta. Fundam. Clin. Pharmacol..

[97] Shabir H., Kundu S., Basir S.F., Khan L.A. (2014). Modulation of Pb(II) caused aortal constriction by eugenol and carvacrol. Biol. Trace Elem. Res..

[98] Koto R., Imamura M., Watanabe C., Obayashi S., Shiraishi M., Sasaki Y., Azuma H. (2006). Linalyl acetate as a major ingredient of lavender essential oil relaxes the rabbit vascular smooth muscle through dephosphorylation of myosin light chain. J. Cardiovasc. Pharmacol..

[99] Johnson C.D., Melanaphy D., Purse A., Stokesberry S.A., Dickson P., Zholos A. (2009). V Transient receptor potential melastatin 8 channel involvement in the regulation of vascular tone. Am. J. Physiol. Hear. Circ. Physiol..

[100] Cheang W.S., Lam M.Y., Wong W.T., Tian X.Y., Lau C.W., Zhu Z., Yao X., Huang Y. (2013). Menthol relaxes rat aortae, mesenteric and coronary arteries by inhibiting calcium influx. Eur. J. Pharmacol..

[101] De Siqueira R.J.B., Freire W.B.S., Vasconcelos-Silva A.A., Fonseca-Magalhães P.A., Lima F.J.B., Brito T.S., Mourão L.T.C., Ribeiro R.A., Lahlou S., Magalhães P.J.C. (2012). In vitro characterization of the pharmacological effects induced by (-)-α-bisabolol in rat smooth muscle preparations. Can. J. Physiol. Pharmacol..

[102] De Siqueira R.J.B., Ribeiro-Filho H.V., Freire R.S., Cosker F., Freire W.B.S., Vasconcelos-Silva A.A., Soares M.A., Lahlou S., Magalhães P.J.C. (2014). (-)-α-Bisabolol inhibits preferentially electromechanical coupling on rat isolated arteries. Vascul. Pharmacol..

[103] Soares P.M.G., Lima R.F., de Freitas Pires A., Souza E.P., Assreuy A.M.S., Criddle D.N. (2007). Effects of anethole and structural analogues on the contractility of rat isolated aorta: Involvement of voltage-dependent Ca^2+^-channels. Life Sci..

[104] Lahlou S., Interaminense L.F., Magalhaes P.J., Leal-Cardoso J.H., Duarte G.P. (2004). Cardiovascular effects of eugenol, a phenolic compound present in many plant essential oils, in normotensive rats. J. Cardiovasc. Pharmacol..

[105] Peixoto-Neves D., Wang Q., Leal-Cardoso J.H., Rossoni L.V., Jaggar J.H. (2015). Eugenol dilates mesenteric arteries and reduces systemic BP by activating endothelial cell TRPV4 channels. Br. J. Pharmacol..

[106] Criddle D.N., Madeira S.V., Soares de Moura R. (2003). Endothelium-dependent and -independent vasodilator effects of eugenol in the rat mesenteric vascular bed. J. Pharm. Pharmacol..

[107] Damiani C.E., Moreira C.M., Zhang H.T., Creazzo T.L., Vassallo D.V. (2004). Effects of eugenol, an essential oil, on the mechanical and electrical activities of cardiac muscle. J. Cardiovasc. Pharmacol..

[108] Damiani C.E.N., Rossoni L.V., Vassallo D.V. (2003). Vasorelaxant effects of eugenol on rat thoracic aorta. Vascul. Pharmacol..

[109] Raffai G., Kim B., Park S., Khang G., Lee D., Vanhoutte P.M. (2014). Cinnamaldehyde and cinnamaldehyde-containing micelles induce relaxation of isolated porcine coronary arteries: Role of nitric oxide and calcium. Int. J. Nanomed..

[110] Yanaga A., Goto H., Nakagawa T., Hikiami H., Shibahara N., Shimada Y. (2006). Cinnamaldehyde induces endothelium-dependent and -independent vasorelaxant action on isolated rat aorta. Biol. Pharm. Bull..

[111] Vasconcelos-Silva A.A., de Lima F.J.B., de Brito T.S., Lahlou S., Magalhães P.J.C. (2014). Vasorelaxation induced by methyl cinnamate, the major constituent of the essential oil of *Ocimum micranthum*, in rat isolated aorta. Clin. Exp. Pharmacol. Physiol..

[112] El Tantawy M.E., El Sakhawy F.S., El Sohly M.A., Ross S.A. (1999). Chemical composition and biological activity of the essential oil of the fruit of *Taxodium distichum* L. rich growing in Egypt. J. Essent. Oil Res..

[113] Reiner Z., Catapano A.L., De Backer G., Graham I., Taskinen M.-R., Wiklund O., Agewall S., Alegria E., Chapman M.J., Durrington P. (2011). ESC/EAS Guidelines for the management of dyslipidaemias: The Task Force for the management of dyslipidaemias of the European Society of Cardiology (ESC) and the European Atherosclerosis Society (EAS). Eur. Heart J..

[114] Ng C.-Y., Leong X.-F., Masbah N., Adam S.K., Kamisah Y., Jaarin K. (2014). Heated vegetable oils and cardiovascular disease risk factors. Vascul. Pharmacol..

[115] Duncan M.S., Vasan R.S., Xanthakis V. (2019). Trajectories of blood lipid concentrations over the adult life course and risk of cardiovascular disease and all-cause mortality: Observations from the Framingham study over 35 years. J. Am. Heart Assoc..

[116] Perk J., De Backer G., Gohlke H., Graham I., Reiner Z., Verschuren M., Albus C., Benlian P., Boysen G., Cifkova R. (2012). European Guidelines on cardiovascular disease prevention in clinical practice (version 2012). The Fifth Joint Task Force of the European Society of Cardiology and other societies on cardiovascular disease prevention in clinical practice. Eur. Heart J..

[117] Kromhout D., Menotti A., Kesteloot H., Sans S. (2002). Prevention of coronary heart disease by diet and lifestyle: Evidence from prospective cross-cultural, cohort, and intervention studies. Circulation.

[118] (2008). Blood Pressure Lowering Treatment Trialists’ Collaboration. Effects of different regimens to lower blood pressure on major cardiovascular events in older and younger adults: Meta-analysis of randomised trials. BMJ.

[119] Law M.R., Morris J.K., Wald N.J. (2009). Use of blood pressure lowering drugs in the prevention of cardiovascular disease: Meta-analysis of 147 randomised trials in the context of expectations from prospective epidemiological studies. BMJ.

[120] Cholesterol Treatment Trialists’ (CTT) Collaborators (2008). Efficacy of cholesterol-lowering therapy in 18 686 people with diabetes in 14 randomised trials of statins: A meta-analysis. Lancet.

[121] Brugts J.J., Yetgin T., Hoeks S.E., Gotto A.M., Shepherd J., Westendorp R.G.J., de Craen A.J.M., Knopp R.H., Nakamura H., Ridker P. (2009). The benefits of statins in people without established cardiovascular disease but with cardiovascular risk factors: Meta-analysis of randomised controlled trials. BMJ.

[122] Rydén L., Grant P.J., Anker S.D., Berne C., Cosentino F., Danchin N., Deaton C., Escaned J., Hammes H.P., Huikuri H. (2013). ESC guidelines on diabetes, pre-diabetes, and cardiovascular diseases developed in collaboration with the EASD. Eur. Heart J..

[123] Racette S.B., Lin X., Lefevre M., Spearie C.A., Most M.M., Ma L., Ostlund R.E. (2010). Dose effects of dietary phytosterols on cholesterol metabolism: A controlled feeding study. Am. J. Clin. Nutr..

[124] Reid I.R., Birstow S.M., Bolland M.J. (2017). Calcium and cardiovascular disease. Endocrinol. Metab..

[125] Marks A.R. (2003). Calcium and the heart: A question of life and death. J. Clin. Investig..

[126] The Emerging Risk Factors Collaboration (2010). Diabetes mellitus, fasting blood glucose concentration, and risk of vascular disease: A collaborative meta-analysis of 102 prospective studies. Lancet.

[127] Singh G.M., Danaei G., Farzadfar F., Stevens G.A., Woodward M., Wormser D., Kaptoge S., Whitlock G., Qiao Q., Lewington S. (2013). The age-specific quantitative effects of metabolic risk factors on cardiovascular diseases and diabetes: A pooled analysis. PLoS ONE.

[128] Haffner S.M., Lehto S., Rönnemaa T., Pyörälä K., Laakso M. (1998). Mortality from coronary heart disease in subjects with type 2 diabetes and in nondiabetic subjects with and without prior myocardial infarction. N. Engl. J. Med..

[129] Sobczak I.S.A., Blindauer A.C., Stewart J.A. (2019). Changes in plasma free fatty acids associated with type-2 diabetes. Nutrients.

[130] Maack C., Lehrke M., Backs J., Heinzel F.R., Hulot J.-S., Marx N., Paulus W.J., Rossignol P., Taegtmeyer H., Bauersachs J. (2018). Heart failure and diabetes: Metabolic alterations and therapeutic interventions: A state-of-the-art review from the Translational Research Committee of the Heart Failure Association–European Society of Cardiology. Eur. Heart J..

[131] Lee M.-H., Chen Y.-Y., Tsai J.-W., Wang S.-C., Watanabe T., Tsai Y.-C. (2011). Inhibitory effect of β-asarone, a component of *Acorus calamus* essential oil, on inhibition of adipogenesis in 3T3-L1 cells. Food Chem..

[132] Shen X.-C., Tao L., Li W.-K., Zhang Y.-Y., Luo H., Xia Y.-Y. (2012). Evidence-based antioxidant activity of the essential oil from Fructus, A. zerumbet on cultured human umbilical vein endothelial cells’ injury induced by ox-LDL. BMC Complement. Altern. Med..

[133] Xiao T., Zeng Y., Xu Y., Zhang Y., Jiang Y., Tao L., Shen X. (2014). The endothelial protective properties of essential oil from Fructus Alpiniae zerumbet via the Akt/NOS-NO signaling pathway in vitro. Planta Med..

[134] Kim J.-H., Lee H.-J., Jeong S.-J., Lee M.-H., Kim S.-H. (2012). Essential oil of *Pinus koraiensis* leaves exerts antihyperlipidemic effects via up-regulation of low-density lipoprotein receptor and inhibition of acyl-coenzyme A: Cholesterol acyltransferase. Phyther. Res..

[135] Chung M.J., Park K.W., Kim K.H., Kim C.-T., Baek J.P., Bang K.-H., Choi Y.-M., Lee S.-J. (2008). Asian plantain (*Plantago asiatica*) essential oils suppress 3-hydroxy-3-methyl-glutaryl-co-enzyme A reductase expression in vitro and in vivo and show hypocholesterolaemic properties in mice. Br. J. Nutr..

[136] Belhadj S., Hentati O., Hammami M., Ben Hadj A., Boudawara T., Dammak M., Zouari S., El Feki A.F. (2018). Metabolic impairments and tissue disorders in alloxan-induced diabetic rats are alleviated by *Salvia officinalis* L. essential oil. Biomed. Pharmacother..

[137] Lima C.F., Azevedo M.F., Araujo R., Fernandes-Ferreira M., Pereira-Wilson C. (2006). Metformin-like effect of *Salvia officinalis* (common sage): Is it useful in diabetes prevention?. Br. J. Nutr..

[138] Kumar S., Vasudeva N., Sharma S. (2012). GC-MS analysis and screening of antidiabetic, antioxidant and hypolipidemic potential of *Cinnamomum tamala* oil in streptozotocin induced diabetes mellitus in rats. Cardiovasc. Diabetol..

[139] Singh V., Jain M., Misra A., Khanna V., Rana M., Prakash P., Malasoni R., Dwivedi A.K., Dikshit M., Barthwal M.K. (2013). Curcuma oil ameliorates hyperlipidaemia and associated deleterious effects in golden Syrian hamsters. Br. J. Nutr..

[140] El-Soud N.A., El-Laithy N., El-Saeed G., Wahby M.S., Khalil M., Morsy F., Shaffie N. (2011). Antidiabetic activities of *Foeniculum vulgare* Mill. essential oil in streptozotocin-induced diabetic rats. Maced. J. Med. Sci..

[141] Al-Okbi S.Y., Hussein A.M.S., Elbakry H.F.H., Fouda K.A., Mahmoud K.F., Hassan M.E. (2018). Health benefits of fennel, rosemary volatile oils and their nano-forms in dyslipidemic rat model. Pak. J. Biol. Sci..

[142] Al-Okbi S.Y., Mohamed D.A., Hamed T.E., Edris A.E. (2014). Protective effect of clove oil and eugenol microemulsions on fatty liver and dyslipidemia as components of metabolic syndrome. J. Med. Food.

[143] Keihan G.S., Gharib M.H., Momeni A., Hemati Z., Sedighin R. (2016). A comparison between the effect of *Cuminum cyminum* and vitamin E on the level of leptin, paraoxonase 1, HbA1c and oxidized LDL in diabetic patients. Int. J. Mol. Cell. Med..

[144] Jafari S., Sattari R., Ghavamzadeh S. (2017). Evaluation the effect of 50 and 100 mg doses of *Cuminum cyminum* essential oil on glycemic indices, insulin resistance and serum inflammatory factors on patients with diabetes type II: A double-blind randomized placebo-controlled clinical trial. J. Tradit. Complement. Med..

[145] Saravanan S., Pari L. (2015). Role of thymol on hyperglycemia and hyperlipidemia in high fat diet-induced type 2 diabetic C57BL/6J mice. Eur. J. Pharmacol..

[146] Ezhumalai M., Ashokkumar N., Pugalendi K.V. (2015). Combination of carvacrol and rosiglitazone ameliorates high fat diet induced changes in lipids and inflammatory markers in C57BL/6J mice. Biochimie.

[147] Galle M., Kladniew B.R., Castro M.A., Villegas S.M., Lacunza E., Polo M., De Bravo M.G., Crespo R. (2015). Modulation by geraniol of gene expression involved in lipid metabolism leading to a reduction of serum-cholesterol and triglyceride levels. Phytomedicine.

[148] Jayachandran M., Chandrasekaran B., Namasivayam N. (2015). Effect of geraniol, a plant derived monoterpene on lipids and lipid metabolizing enzymes in experimental hyperlipidemic hamsters. Mol. Cell. Biochem..

[149] Vallianou I., Peroulis N., Pantazis P., Hadzopoulou-Cladaras M. (2011). Camphene, a plant-derived monoterpene, reduces plasma cholesterol and triglycerides in hyperlipidemic rats independently of HMG-CoA reductase activity. PLoS ONE.

[150] Naderi G.A., Asgary S., Ani M., Sarraf-Zadegan N., Safari M.R. (2004). Effect of some volatile oils on the affinity of intact and oxidized low-density lipoproteins for adrenal cell surface receptors. Mol. Cell. Biochem..

[151] Basha R.H., Sankaranarayanan C. (2016). β-Caryophyllene, a natural sesquiterpene lactone attenuates hyperglycemia mediated oxidative and inflammatory stress in experimental diabetic rats. Chem. Biol. Interact..

[152] Kumawat V.S., Kaur G. (2020). Insulinotropic and antidiabetic effects of β-caryophyllene with L-arginine in type 2 diabetic rats. J. Food Biochem..

[153] Basha R.H., Sankaranarayanan C. (2015). Protective role of β-caryophyllene, a sesquiterpene lactone on plasma and tissue glycoprotein components in streptozotocin-induced hyperglycemic rats. J. Acute Med..

[154] Youssef D.A., El-Fayoumi H.M., Mahmoud M.F. (2019). Beta-caryophyllene protects against diet-induced dyslipidemia and vascular inflammation in rats: Involvement of CB2 and PPAR-γ receptors. Chem. Biol. Interact..

[155] Youssef D.A., El-Fayoumi H.M., Mahmoud M.F. (2019). Beta-caryophyllene alleviates diet-induced neurobehavioral changes in rats: The role of CB2 and PPAR-γ receptors. Biomed. Pharmacother..

[156] Basha R.H., Sankaranarayanan C. (2014). β-Caryophyllene, a natural sesquiterpene, modulates carbohydrate metabolism in streptozotocin-induced diabetic rats. Acta Histochem..

[157] Suijun W., Zhen Y., Ying G., Yanfang W. (2014). A role for trans-caryophyllene in the moderation of insulin secretion. Biochem. Biophys. Res. Commun..

[158] Baddar N.W.A.-H., Aburjai T.A., Taha M.O., Disi A.M. (2011). Thujone corrects cholesterol and triglyceride profiles in diabetic rat model. Nat. Prod. Res..

[159] Alkhateeb H., Al-Duais M., Qnais E., Trad B., Matalgah L. (2018). Plasma glucose-lowering effect of thujone and its molecular mechanisms of action in streptozotocin-induced diabetic rats. Pharmacol. Online.

[160] Alkhateeb H.H. (2015). Thujone improves glucose homeostasis in streptozotocin-induced diabetic rats through activation of Akt/glycogen synthase kinase-3β signaling pathway. J. Exp. Integr. Med..

[161] Chellian R., Pandy V., Mohamed Z. (2017). Pharmacology and toxicology of α- and β-asarone: A review of preclinical evidence. Phytomedicine.

[162] Thakare M.M., Surana S.J. (2016). β-Asarone modulate adipokines and attenuates high fat diet-induced metabolic abnormalities in Wistar rats. Pharmacol. Res..

[163] Poplawski J., Lozowicka B., Dubis A.T., Lachowska B., Witkowski S., Siluk D., Petrusewicz J., Kaliszan R., Cybulski J., Strzalkowska M. (2000). Synthesis and hypolipidemic and antiplatelet activities of α-asarone isomers in humans (in vitro), mice (in vivo), and rats (in vivo). J. Med. Chem..

[164] Venkadeswaran K., Thomas P.A., Geraldine P. (2016). An experimental evaluation of the anti-atherogenic potential of the plant, *Piper betle*, and its active constitutent, eugenol, in rats fed an atherogenic diet. Biomed. Pharmacother..

[165] Venkadeswaran K., Muralidharan A.R., Annadurai T., Ruban V.V., Sundararajan M., Anandhi R., Thomas P.A., Geraldine P. (2014). Antihypercholesterolemic and antioxidative potential of an extract of the plant, *Piper betle*, and its active constituent, eugenol, in Triton WR-1339-induced hypercholesterolemia in experimental rats. Evid. Based. Complement. Alternat. Med..

[166] Wang F., Pu C., Zhou P., Wang P., Liang D., Wang Q., Hu Y., Li B., Hao X. (2015). Cinnamaldehyde prevents endothelial dysfunction induced by high glucose by activating Nrf2. Cell. Physiol. Biochem..

[167] Willoughby S., Holmes A., Loscalzo J. (2002). Platelets and cardiovascular disease. Eur. J. Cardiovasc. Nurs..

[168] Carobbio A., Thiele J., Passamonti F., Rumi E., Ruggeri M., Rodeghiero F., Randi M.L., Bertozzi I., Vannucchi A.M., Antonioli E. (2011). Risk factors for arterial and venous thrombosis in WHO-defined essential thrombocythemia: An international study of 891 patients. Blood.

[169] Gawaz M. (2004). Role of platelets in coronary thrombosis and reperfusion of ischemic myocardium. Cardiovasc. Res..

[170] Schanze N., Bode C., Duerschmied D. (2019). Platelet contributions to myocardial ischemia/reperfusion injury. Front. Immunol..

[171] Uhl K., Shuldiner A.R., Klein T.E., Altman R.B. (2011). Platelet aggregation pathway. Pharmacogenet. Genomics.

[172] Tognolini M., Barocelli E., Ballabeni V., Bruni R., Bianchi A., Chiavarini M., Impicciatore M. (2006). Comparative screening of plant essential oils: Phenylpropanoid moiety as basic core for antiplatelet activity. Life Sci..

[173] Yan Y.O.W., Zhao X., Ye X., Zhang C., Hao J., He J., Zhu X., Xu H., Yang X. (2010). Effect of essential oil of *Syringa pinnatifolia* Hemsl. var. *alashanensis* on ischemia of myocardium, hypoxia and platelet aggregation. J. Ethnopharmacol..

[174] Chang M.C., Uang B.J., Tsai C.Y., Wu H.L., Lin B.R., Lee C.S., Chen Y.J., Chang C.H., Tsai Y.L., Kao C.J. (2007). Hydroxychavicol, a novel betel leaf component, inhibits platelet aggregation by suppression of cyclooxygenase, thromboxane production and calcium mobilization. Br. J. Pharmacol..

[175] You J.H., Kang P., Min S.S., Seol G.H. (2013). Bergamot essential oil differentially modulates intracellular Ca^2+^ Levels in vascular endothelial and smooth muscle cells. J. Cardiovasc. Pharmacol..

[176] Maiwulanjiang M., Bi C.W.C., Lee P.S.C., Xin G., Miernisha A., Lau K.M., Xiong A., Li N., Dong T.T.X., Aisa H.A. (2015). The volatile oil of Nardostachyos Radix et Rhizoma induces endothelial nitric oxide synthase activity in HUVEC cells. PLoS ONE.

[177] Magyar J., Szentandrássy N., Bányász T., Fülöp L., Varró A., Nánási P.P. (2004). Effects of terpenoid phenol derivatives on calcium current in canine and human ventricular cardiomyocytes. Eur. J. Pharmacol..

[178] Magyar J., Szentandrássy N., Bányász T., Fülöp L., Varró A., Nánási P.P. (2002). Effects of thymol on calcium and potassium currents in canine and human ventricular cardiomyocytes. Br. J. Pharmacol..

[179] Szentandrássy N., Szigeti G., Szegedi C., Sárközi S., Magyar J., Bányász T., Csernoch L., Kovács L., Nánási P.P., Jóna I. (2004). Effect of thymol on calcium handling in mammalian ventricular myocardium. Life Sci..

[180] Earley S., Gonzales A.L., Garcia Z.I. (2010). A dietary agonist of transient receptor potential cation channel V3 elicits endothelium-dependent vasodilation. Mol. Pharmacol..

[181] Sensch O., Vierling W., Brandt W., Reiter M. (2000). Effects of inhibition of calcium and potassium currents in guinea-pig cardiac contraction: Comparison of β-caryophyllene oxide, eugenol, and nifedipine. Br. J. Pharmacol..

[182] Alvarez-Collazo J., Alonso-Carbajo L., López-Medina A.I., Alpizar Y.A., Tajada S., Nilius B., Voets T., López-López J.R., Talavera K., Pérez-García M.T. (2014). Cinnamaldehyde inhibits L-type calcium channels in mouse ventricular cardiomyocytes and vascular smooth muscle cells. Pflugers Arch..

[183] Nascimento N.R.F., Leal-Cardoso J.H., Lessa L.M.A., Roriz-Filho J.S., Cunha K.M.A., Fonteles M.C. (2005). Terpinen-4-ol: Mechanisms of relaxation on rabbit duodenum. J. Pharm. Pharmacol..

[184] Astudillo A., Hong E., Bye R., Navarrete A. (2004). Antispasmodic activity of extracts and compounds of *Acalypha phleoides* Cav. Phytother. Res..

[185] Danesi F., Elementi S., Neri R., Maranesi M., D’Antuono L.F., Bordoni A. (2008). Effect of cultivar on the protection of cardiomyocytes from oxidative stress by essential oils and aqueous extracts of basil (*Ocimum basilicum* L.). J. Agric. Food Chem..

[186] Szűcs G., Murlasits Z., Török S., Kocsis G.F., Pálóczi J., Görbe A., Csont T., Csonka C., Ferdinandy P. (2013). Cardioprotection by farnesol: Role of the mevalonate pathway. Cardiovasc. Drugs Ther..

[187] Lee K.P., Sudjarwo G.W., Jung S.H., Lee D., Lee D.Y., Lee G.B., Baek S., Kim D.Y., Lee H.M., Kim B. (2015). Carvacrol inhibits atherosclerotic neointima formation by downregulating reactive oxygen species production in vascular smooth muscle cells. Atherosclerosis.

[188] Liu F., Huang Z.-Z., Sun Y.-H., Li T., Yang D.-H., Xu G., Su Y.-Y., Zhang T. (2017). Four main active ingredients derived from a Traditional Chinese Medicine Guanxin Shutong capsule cause cardioprotection during myocardial ischemia injury calcium overload suppression. Phyther. Res..

[189] Fouad A.A., Yacoubi M.T. (2011). Mechanisms underlying the protective effect of eugenol in rats with acute doxorubicin cardiotoxicity. Arch. Pharm. Res..

[190] Mnafgui K., Hajji R., Derbali F., Gammoudi A., Khabbabi G., Ellefi H., Allouche N., Kadri A., Gharsallah N. (2016). Anti-inflammatory, antithrombotic and cardiac remodeling preventive effects of eugenol in isoproterenol-induced myocardial infarction in Wistar rat. Cardiovasc. Toxicol..

[191] Song F., Li H., Sun J., Wang S. (2013). Protective effects of cinnamic acid and cinnamic aldehyde on isoproterenol-induced acute myocardial ischemia in rats. J. Ethnopharmacol..

[192] Yang L., Wu Q.-Q., Liu Y., Hu Z.-F., Bian Z.-Y., Tang Q.-Z. (2015). Cinnamaldehyde attenuates pressure overload-induced cardiac hypertrophy. Int. J. Clin. Exp. Pathol..

[193] Zhao H., Zhang M., Zhou F., Cao W., Bi L., Xie Y., Yang Q., Wang S. (2016). Cinnamaldehyde ameliorates LPS-induced cardiac dysfunction via TLR4-NOX4 pathway: The regulation of autophagy and ROS production. J. Mol. Cell. Cardiol..

[194] Shi H.-X., Yang J., Yang T., Xue Y.-L., Liu J., Li Y.-J., Zhang D.-D., Xu J.-W., Bian K. (2014). Alpha-asarone protects endothelial cells from injury by angiotensin II. Evid. Based Complement. Altern. Med..

